# Human Cytomegalovirus Immediate-Early 1 Protein Rewires Upstream STAT3 to Downstream STAT1 Signaling Switching an IL6-Type to an IFNγ-Like Response

**DOI:** 10.1371/journal.ppat.1005748

**Published:** 2016-07-07

**Authors:** Thomas Harwardt, Simone Lukas, Marion Zenger, Tobias Reitberger, Daniela Danzer, Theresa Übner, Diane C. Munday, Michael Nevels, Christina Paulus

**Affiliations:** 1 Institute for Medical Microbiology and Hygiene, University of Regensburg, Regensburg, Germany; 2 Biomedical Sciences Research Complex, University of St Andrews, St Andrews, United Kingdom; University of Wisconsin-Madison, UNITED STATES

## Abstract

The human cytomegalovirus (hCMV) major immediate-early 1 protein (IE1) is best known for activating transcription to facilitate viral replication. Here we present transcriptome data indicating that IE1 is as significant a repressor as it is an activator of host gene expression. Human cells induced to express IE1 exhibit global repression of IL6- and oncostatin M-responsive STAT3 target genes. This repression is followed by STAT1 phosphorylation and activation of STAT1 target genes normally induced by IFNγ. The observed repression and subsequent activation are both mediated through the same region (amino acids 410 to 445) in the C-terminal domain of IE1, and this region serves as a binding site for STAT3. Depletion of STAT3 phenocopies the STAT1-dependent IFNγ-like response to IE1. In contrast, depletion of the IL6 receptor (IL6ST) or the STAT kinase JAK1 prevents this response. Accordingly, treatment with IL6 leads to prolonged STAT1 instead of STAT3 activation in wild-type IE1 expressing cells, but not in cells expressing a mutant protein (IE1dl410-420) deficient for STAT3 binding. A very similar STAT1-directed response to IL6 is also present in cells infected with a wild-type or revertant hCMV, but not an IE1dl410-420 mutant virus, and this response results in restricted viral replication. We conclude that IE1 is sufficient and necessary to rewire upstream IL6-type to downstream IFNγ-like signaling, two pathways linked to opposing actions, resulting in repressed STAT3- and activated STAT1-responsive genes. These findings relate transcriptional repressor and activator functions of IE1 and suggest unexpected outcomes relevant to viral pathogenesis in response to cytokines or growth factors that signal through the IL6ST-JAK1-STAT3 axis in hCMV-infected cells. Our results also reveal that IE1, a protein considered to be a key activator of the hCMV productive cycle, has an unanticipated role in tempering viral replication.

## Author Summary

Our previous work has shown that the human cytomegalovirus (hCMV) major immediate-early 1 protein (IE1) modulates host cell signaling pathways involving proteins of the signal transducer and activator of transcription (STAT) family. IE1 has also long been known to facilitate viral replication by activating transcription. In this report we demonstrate that IE1 is as significant a repressor as it is an activator of host gene expression. Many genes repressed by IE1 are normally induced via STAT3 signaling triggered by interleukin 6 (IL6) or related cytokines, whereas many genes activated by IE1 are normally induced via STAT1 signaling triggered by interferon gamma (IFNγ). Our results suggest that the repression of STAT3- and the activation of STAT1-responsive genes by IE1 are coupled. By targeting STAT3, IE1 rewires upstream STAT3 to downstream STAT1 signaling. Consequently, genes normally induced by IL6 are repressed while genes normally induced by IFNγ become responsive to IL6 in the presence of IE1. We also demonstrate that, by switching an IL6 to an IFNγ-like response, IE1 tempers viral replication. These results suggest an unanticipated dual role for IE1 in either promoting or limiting hCMV propagation and demonstrate how a key viral regulatory protein merges two central cellular signaling pathways to divert cytokine responses relevant to hCMV pathogenesis.

## Introduction

Janus kinase-signal transducer and activator of transcription (JAK-STAT) signaling pathways are the principal means by which responses to dozens of cytokines, growth factors and other extracellular molecules are transduced from the cell surface to the nucleus. Although all JAK-STAT pathways share the same design principle, they involve distinct sets of ligands that engage different receptor and effector components to activate groups of genes which only partly overlap [1, 2].

For interleukin (IL) 6 family cytokines, including IL6 and oncostatin M (OSM), JAK-STAT pathway activation begins with ligand binding to specific receptors, such as the IL6 receptor (IL6Rα or IL6R) and the OSM receptor, respectively. The ligand-receptor interaction is followed by dimerization of the IL6 signal transducer (IL6Rβ, GP130 or IL6ST) subunits common to all IL6 family cytokine receptors. IL6ST is constitutively associated with several JAK family tyrosine kinases (JAK1, JAK2 and TYK2) of which JAK1 seems to be the most important for signaling in response to IL6 [3, 4]. Upon receptor activation, JAK1 is phosphorylated and the activated kinase subsequently phosphorylates tyrosine residues in the cytoplasmic tail of IL6ST. These phosphotyrosines serve as docking sites for the src homology 2 (SH2) domain of cytoplasmic STAT3. Following recruitment to the receptor, STAT3 is phosphorylated on a single tyrosine residue (Y705) by JAK1 or other kinases. Y705 phosphorylation is required for the formation of functional STAT3 dimers (typically homodimers) through reciprocal SH2-phosphotyrosyl interactions. The active pSTAT3 dimers subsequently dissociate from the receptor and accumulate in the nucleus, most likely coordinate with their ability to bind DNA [5]. DNA binding occurs rather sequence-specifically, resulting in transcriptional activation of select target genes involved in diverse processes including cell survival and proliferation [1, 6, 7]. One of the pSTAT3 target genes encodes the suppressor of cytokine signaling 3 (SOCS3) which forms part of a negative feedback circuit by inhibiting IL6 signaling [8, 9].

Another group of cytokines, the interferons (IFNs), are distinct from the IL6-type cytokines but also trigger signaling through JAK-STAT pathways. For type I IFNs, including IFNα and IFNβ, canonical signaling occurs through the IFNα/β receptor subunits (IFNAR1 and IFNAR2), JAK1 and TYK2, and a trimeric complex of tyrosine-phosphorylated STAT1 and STAT2 with IFN regulatory factor 9 (IRF9). This complex is also referred to as IFN-stimulated gene factor 3 (ISGF3) and activates transcription of numerous genes many of which encode anti-viral products [10, 11]. For IFNγ, the only type II IFN, signaling is typically mediated via the IFNγ receptor (IFNGR) subunits 1 and 2, JAK1 and JAK2, and tyrosine (Y701)-phosphorylated STAT1 (pSTAT1) homodimers. Like other STAT proteins, STAT1 also undergoes serine (S727) phosphorylation adding to its potency as a transcriptional activator. By triggering prolonged STAT1 activation, type II IFN signaling results in the induction of numerous genes broadly defined as immune-modulatory [12, 13].

The pathways responsive to IL6-type cytokines or IFNγ share important intracellular signaling molecules, but have been linked to opposing actions. STAT3 generally promotes cell survival and proliferation, may counteract inflammation and induces immune tolerance. In contrast, STAT1 tends to promote apoptosis, inhibits proliferation and favors innate or adaptive immune responses. Due to cross-regulation between the two pathways, perturbations in the levels or activities of STAT1 and STAT3 may redirect cytokine signals with unexpected outcomes [14–17].

Many viruses target components of JAK-STAT pathways including STAT1, STAT2 and STAT3. STAT1 and STAT2 usually act anti-viral due to their essential roles in IFN signaling [10, 12]. Accordingly, most viruses antagonize STAT1 or STAT2 [18, 19], although viral activation and annexation of STAT1 has also been reported [20–28]. For STAT3, the role in viral infections appears to be more complex. Thus, viruses either positively or negatively affect the expression or activity of STAT3 [29–36].

Human cytomegalovirus (hCMV), one of eight human herpesviruses, is a very widespread opportunistic pathogen. To accomplish efficient replication and lasting persistence, hCMV seems to tweak most, if not all, host cell signaling pathways [37–40]. The 72-kDa (491 amino-acid) immediate-early 1 protein (IE1) has emerged as hCMV’s key modulator of JAK-STAT signaling [41–46]. Following infection of permissive cells, IE1 is among the very first and most abundant gene products produced *de novo* from the hCMV genome. The viral protein accumulates in the host cell nucleus and sets the stage for efficient hCMV early gene expression and subsequent viral replication [47–51]. The first hint suggesting IE1 may impact JAK-STAT pathways came from our finding that the protein confers increased type I IFN resistance to hCMV without negatively affecting IFN expression [52]. This phenotype was partly attributed to nuclear complex formation between IE1 and STAT2 depending on amino acids 373 to 445 [53] or 421 to 475 [54] in the viral protein’s C-terminal domain (amino acids 373 to 491). This domain is thought to be structurally largely disordered and contains four patches with highly biased amino acid composition: three acidic ‘domains’ (AD1-AD3) and one serine/proline-rich stretch (S/P) [41, 53, 55]. The sequences downstream from the STAT2 interaction site in the C-terminal domain of IE1 feature a small ubiquitin-like modifier (SUMO) conjugation motif (amino acids 449–452) [56–58] and a chromatin tethering domain (CTD, amino acids 476–491) [59–61] which mediate binding to SUMO1 and to the acidic pocket formed by histones H2A-H2B on the nucleosome surface [62], respectively. SUMOylation of IE1 may negatively regulate STAT2 binding [54] and positively affect hCMV replication [58]. IE1-STAT2 interaction causes diminished sequence-specific DNA binding by ISGF3 and inhibited type I ISG activation in the presence of IFNα or IFNβ [52–54, 63]. The viral protein’s ability to inhibit type I ISG induction via STAT2 interaction is believed to be important, because it contributes to efficient hCMV replication [53, 54] and appears to be conserved across IE1 homologs of the β-herpesvirus subfamily [64]. Besides functioning as an antagonist of type I IFN signaling, IE1 can also act as an agonist of type II IFN signaling. Following expression under conditions mimicking the situation during hCMV infection, IE1 elicited a host transcriptional response dominated by the up-regulation of genes normally induced by IFNγ. The IE1-dependent gene activation proved to be independent of IFNγ and other IFNs, yet required the Y701-phosphorylated form of STAT1. Accordingly, IE1 induced Y701 and S727 phosphorylation, nuclear accumulation and binding of STAT1 to type II ISG promoters [21]. Whether IE1 binds to STAT1 directly or only indirectly (via STAT heterodimers) has not been resolved. Finally, STAT3 was shown to physically interact with IE1, most likely via direct binding. The functional consequences of this interaction include STAT3 nuclear accumulation, disruption of IL6-induced STAT3 Y705 phosphorylation and inhibition of STAT3 binding to the SOCS3 promoter. These events are followed by diminished STAT3-dependent SOCS3 induction upon hCMV infection or IE1 expression [30] adding to the emerging evidence for transcriptional repression by the viral protein. However, IE1 has mostly been recognized as an activator of cellular and viral gene expression [42, 65] and, to the best of our knowledge, no genome-wide analysis of human genes repressed by the viral protein has been pursued.

Here we show, based on genome-wide transcriptome data, that IE1 is as much a repressor as it is an activator of human gene expression. We further demonstrate that a single motif (amino acids 410 to 420) in the C-terminal domain of IE1 links the viral protein’s repressor and activator functions by rewiring upstream IL6-type to downstream IFNγ-like signaling resulting in repressed STAT3- and activated STAT1-responsive genes. Finally, the diversion of STAT3/STAT1 signaling attenuates viral replication revealing an unanticipated temperance activity in IE1.

## Results

### IE1 is as significant a repressor as it is an activator of host gene expression

**Fig 1:**
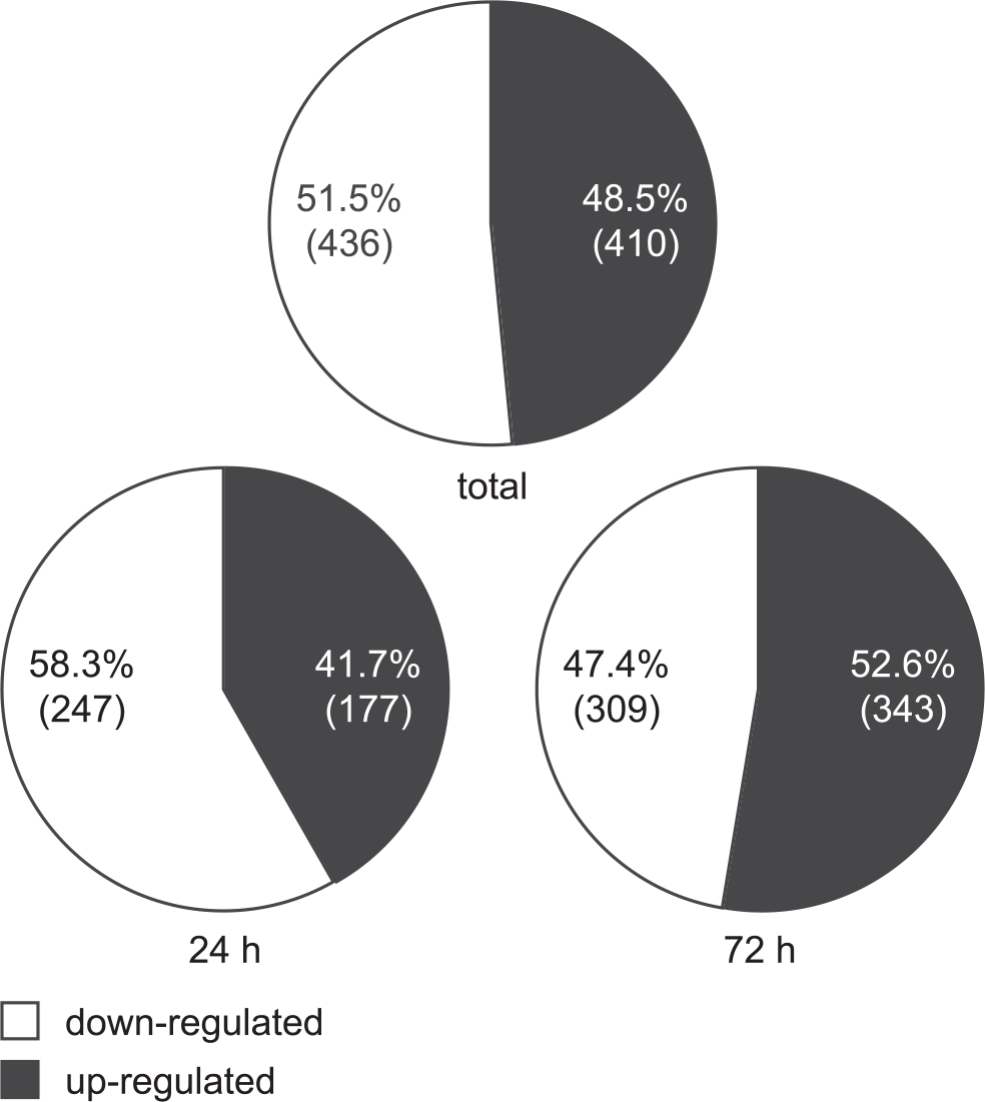
IE1 is both an activator and a repressor of human gene expression. Relative proportions and absolute counts (in brackets) of unique probe sets significantly up- or down-regulated in GeneChip analyses following induction of IE1 for 24 h, 72 h or 24 h and 72 h combined (total). The numbers are based on S1 Data.

We previously reported on an Affymetrix GeneChip analysis of human transcripts undergoing up-regulation in MRC-5 cells transduced to express doxycycline (dox)-inducible IE1 (TetR-IE1 cells). Expression from the preponderant majority (>98%) of genes represented on the GeneChips was not significantly affected by IE1. However, a set of genes were specifically and reproducibly up-regulated by the viral protein [21]. Upon further inspection of the GeneChip data, we noticed that the IE1-dependent changes in the human transcriptome were not biased towards activation. Instead, the numbers of genes significantly up- or down-regulated by IE1 were roughly the same (410 up-regulated and 436 down-regulated probe sets). Notably, at 24 h post IE1 induction there were fewer (<42%) up-regulated compared to down-regulated genes, whereas after 72 h of IE1 expression the up-regulated (>52%) slightly outbalanced the down-regulated genes (Fig 1 and S1 Data).

These data indicate that IE1 is not only an activator, but also a significant repressor of host gene transcription.

### IE1 down-regulates IL6- and OSM-responsive pSTAT3 target genes

Our previous study has shown that the genes activated by IE1 in the TetR-IE1 cell model are largely responsive to pSTAT1 and IFNγ [21]. To identify upstream regulators common to genes repressed by IE1, we used Ingenuity Pathway Analysis. Among probe sets exhibiting negative fold changes of ≥1.5, this analysis identified highly significant associations with STAT3 (p-value of overlap with reference set = 2.5×10^−13^), OSM (p = 6.2×10^−9^), IL6 (p = 1.2×10^−7^) and other components of STAT3-dependent signaling pathways including cytokines (e.g., leukemia inhibitory factor [LIF], p = 8.2×10^−8^, and granulocyte colony-stimulating factor [GCSF], p = 8.4×10^−7^), growth factors (e.g., hepatocyte growth factor, p = 2.2×10^−5^, and epidermal growth factor, p = 2.6×10^−4^) and receptors (e.g., IL10 receptor α, p = 1.2×10^−6^, and IL6ST, p = 1.2×10^−4^). Consistently, 50 genes known to be positively regulated by STAT3, IL6 or/and OSM were identified among the IE1-repressed genes based on Ingenuity Pathway Analysis (Table 1). Moreover, the majority of genes most significantly repressed by IE1 are known STAT3 targets (Table 2).

**Table 1:** Ingenuity analysis^1^ of human genes repressed by IE1 and activated by STAT3, IL6 or OSM.

STAT3^2^				IL6^3^				OSM^4^			
Probe^5^	Gene^6^	Change^7^	Ref.^8^	Probe^5^	Gene^6^	Change^7^	Ref.^8^	Probe^5^	Gene^6^	Change^7^	Ref.^8^
8092169	TNFSF10	-3.1	[81]	8018864	**SOCS3**	-3.0	[125]	8018864	**SOCS3**	-3.0	[126]
8018864	**SOCS3**	-3.0	[127]	8062461	LBP	-2.9	[128]	8062461	LBP	-2.9	[97]
8062461	LBP	-2.9	[129]	8100393	KDR	-2.7	[130]	7898057	PDPN	-2.5	[12]
8100393	KDR	-2.7	[131]	8095744	AREG	-2.4	[132]	7933194	**CXCL12**	-2.2	[133]
7923547	CHI3L1	-2.5	[134]	7902527	**PTGFR**	-2.1	[135]	7903920	CHI3L2	-2.1	[136]
8095744	AREG	-2.4	[132]	8043995	IL1R1	-2.1	[137]	8097991	TDO2	-2.1	[138]
7933194	**CXCL12**	-2.2	[81]	8114612	CD14	-2.1	[139]	8008454	ABCC3	-2.0	[140]
8141016	TFPI2	-2.2	[133]	8008454	ABCC3	-2.0	[140]	8069689	ADAMTS5	-2.0	[141]
7903393	S1PR1	-2.1	[142]	8069689	ADAMTS5	-2.0	[143]	8091411	TM4SF1	-2.0	[136]
7937335	IFITM1	-2.1	[81]	8092691	BCL6	-2.0	[144]	8069676	ADAMTS1	-1.9	[141]
8043995	IL1R1	-2.1	[145]	8004510	CD68	-1.9	[146]	8105040	OSMR	-1.9	[147]
7971015	SMAD9	-2.0	[82]	8069676	ADAMTS1	-1.9	[148]	8151447	IL7	-1.9	[149]
8092691	BCL6	-2.0	[150]	8136557	TBXAS1	-1.9	[151]	8122365	ADGRG6	-1.8	[12]
8151447	IL7	-1.9	[152]	8149927	CLU	-1.9	[153]	7933084	NAMPT	-1.6	[154]
7906400	**IFI16**	-1.8	[155]	8151447	IL7	-1.9	[156]	8015607	STAT3	-1.6	[157]
7924987	AGT	-1.8	[158]	7906400	**IFI16**	-1.8	[158]	8123598	SERPINB1	-1.6	[136]
8055952	NR4A2	-1.8	[159]	7924987	AGT	-1.8	[160]	8140556	HGF	-1.6	[133]
7905789	IL6R	-1.7	[161]	8152522	ENPP2	-1.8	[162]	8156199	DAPK1	-1.6	[136]
7945371	IFITM3	-1.7	[163]	7905789	IL6R	-1.7	[164]	7926875	BAMBI	-1.5	[136]
8130556	SOD2	-1.7	[165]	7945371	IFITM3	-1.7	[160]	8061564	ID1	-1.5	[166]
8174598	IL13RA2	-1.7	[132]	8130556	SOD2	-1.7	[167]	8150592	CEBPD	-1.5	[97]
7933084	NAMPT	-1.6	[154]	7933084	NAMPT	-1.6	[154]	8169580	IL13RA1	-1.5	[136]
7937330	IFITM2	-1.6	[168]	8015607	STAT3	-1.6	[157]				
8015607	STAT3	-1.6	[169]	8140556	HGF	-1.6	[170]				
8065071	FLRT3	-1.6	[81]	7897877	TNFRSF1B	-1.5	[171]				
8123598	SERPINB1	-1.6	[81]	7905047	FCGR1A	-1.5	[172]				
8140556	HGF	-1.6	[173]	8061564	ID1	-1.5	[174]				
7897877	TNFRSF1B	-1.5	[175]	8119161	PIM1	-1.5	[176]				
7905047	FCGR1A	-1.5	[177]	8150592	CEBPD	-1.5	[97]				
8119161	PIM1	-1.5	[178]								
8150592	CEBPD	-1.5	[179]								

^1^ Ingenuity Pathway Analysis (Core Analysis, default analysis settings) of all down-regulated probe sets from S1 Data.

^2^ STAT3 target genes identified by Ingenuity Upstream Analysis.

^3^ IL6 target genes identified by Ingenuity Upstream Analysis.

^4^ OSM target genes identified by Ingenuity Upstream Analysis.

^5^ Affymetrix Probe Set ID.

^6^ Gene Symbol (National Center for Biotechnology Information); genes further pursued in this work are bold-typed.

^7^ Maximum average fold change in comparisons of dox-treated TetR-IE1 vs. dox-treated TetR or dox-treated TetR-IE1 vs. solvent-treated TetR-IE1 cells 24 h or 72 h post induction.

^8^ Key reference.

**Table 2:** Human genes most significantly repressed by IE1^1^.

Probe Set ID	Gene Symbol	24 h post induction^2^		72 h post induction^3^		STAT3-responsive^4^
		IE1+/TetR+	IE1+/IE1-	IE1+/TetR+	IE1+/IE1-	
8077270	**CHL1**	-2.8	-1.5	-5.5	-2.8	Fig 2
8005048	**MYOCD**	-3.9	-1.7	-4.2	-1.9	S1 Fig
8141094	**PDK4**	-3.8	-1.3	-3.5	-1.4	S1 Fig
8156706	**TMOD1**	-1.9	-1.5	-3.3	-2.6	S1 Fig
7929511	ENTPD1	-2.5	-1.2	-3.1	-1.5	[180]
8018864	**SOCS3**	-2.8	-2.1	-3.0	-2.5	Fig 2
8095585	SLC4A4	-1.7	-1.2	-3.0	-2.1	[180]
8077366	**LRRN1**	-2.1	1.1	-3.0	-1.1	S1 Fig
8062461	LBP	-2.1	-1.2	-2.9	-1.5	[180]
7925929	**AKR1C3**	-2.4	-1.6	-2.8	-2.2	S1 Fig
7921916	**RGS5**	-2.8	-1.1	-2.8	1.0	S1 Fig
8178712	TNXB	-1.6	-1.2	-2.7	-2.0	[82]
8179935	TNXB	-1.6	-1.2	-2.7	-2.0	[82]
8092169	TNFSF10	-3.1	-1.2	-2.7	1.0	[81]
8149825	**STC1**	-2.0	1.0	-2.7	-1.5	—^5^
8166632	GK	-2.4	-1.7	-2.6	-1.7	[81]
8101788	UNC5C	-2.4	-1.1	-2.6	-1.4	[180]
8174103	GK	-2.4	-1.7	-2.5	-1.7	[81]
7923547	CHI3L1	-1.1	-1.2	-2.5	-2.4	[134]
7898057	PDPN	-2.2	1.0	-2.5	-1.1	[180]
7968236	**RASL11A**	-2.1	-1.6	-2.4	-2.2	Fig 2
8100109	GABRA2	-2.6	-1.2	-2.4	-1.1	[180]
8118409	**C4A**	-1.4	-1.2	-2.4	-2.2	Fig 2
8118455	**C4A**	-1.4	-1.2	-2.4	-2.2	Fig 2
8179399	**C4A**	-1.4	-1.2	-2.4	-2.2	Fig 2
7972157	**EDNRB**	-3.3	-1.2	-2.4	1.0	S1 Fig
8063437	TSHZ2	-2.5	-1.3	-2.3	-1.3	[180]
8057486	**PDE1A**	-1.3	-1.3	-2.3	-2.3	S1 Fig
7918857	**TSPAN2**	-3.2	1.0	-2.3	1.5	S1 Fig
8056376	SCN3A	-1.2	-1.6	-2.2	-2.7	[180]
8063444	TSHZ2	-2.6	-1.3	-2.2	-1.2	[180]
8064868	**GPCPD1**	-2.7	-1.1	-2.2	-1.2	S1 Fig
8045539	KYNU	-2.6	-1.2	-2.2	-1.1	[81]
7933194	**CXCL12**	-1.2	-1.2	-2.2	-2.2	Fig 2
7908376	**RGS18**	-3.6	-1.3	-2.2	1.0	S1 Fig
7902527	**PTGFR**	-1.7	-1.6	-2.1	-2.1	—^5^ ^,^ ^6^
8166925	MAOA	-2.8	-1.2	-2.1	1.0	[180]
8151942	**HRSP12**	-1.3	-1.3	-2.0	-2.0	S1 Fig
7985032	(LOC391532)^7^	-2.2	-2.6	1.4	-1.2	ND
*7906400*	***IFI16***	*-1*.*3*	*-1*.*4*	*-1*.*7*	*-1*.*8*	*Fig 2*

^1^ ≥2.0-fold average decrease in at least one or ≥1.5-fold average decrease in at least two of the following four comparisons: dox-treated TetR-IE1 (TetR-IE1+) vs. dox-treated TetR (TetR+) cells 24 h post induction, TetR-IE1+ vs. TetR+ cells 72 h post induction, TetR-IE1+ vs. solvent-treated TetR-IE1 (TetR-IE1-) cells 24 h post induction or TetR-IE1+ vs. TetR-IE1- cells 72 h post induction; and ≤1.5-fold average decrease in comparisons of TetR+ vs. TetR cells 24 h or 72 h post induction. Symbols of genes further investigated in this work are bold-typed. IFI16 was further investigated despite narrowly missing the criteria outlined above.

^2^ maximum average fold change in comparisons of TetR-IE1+ vs. TetR+ or TetR-IE1+ vs. TetR-IE1- cells 24 h post induction.

^3^ maximum average fold change in comparisons of TetR-IE1+ vs. TetR+ or TetR-IE1+ vs. TetR-IE1- cells 72 h post induction.

^4^ known target of STAT3 up-regulation or/and binding based on provided reference or this work (Fig 2, S1 Fig); ND, not determined.

^5^ not significantly STAT3-responsive according to S1 Fig.

^6^ IL6-responsive according to Table 1.

^7^ long non-coding RNA.

**Fig 2:**
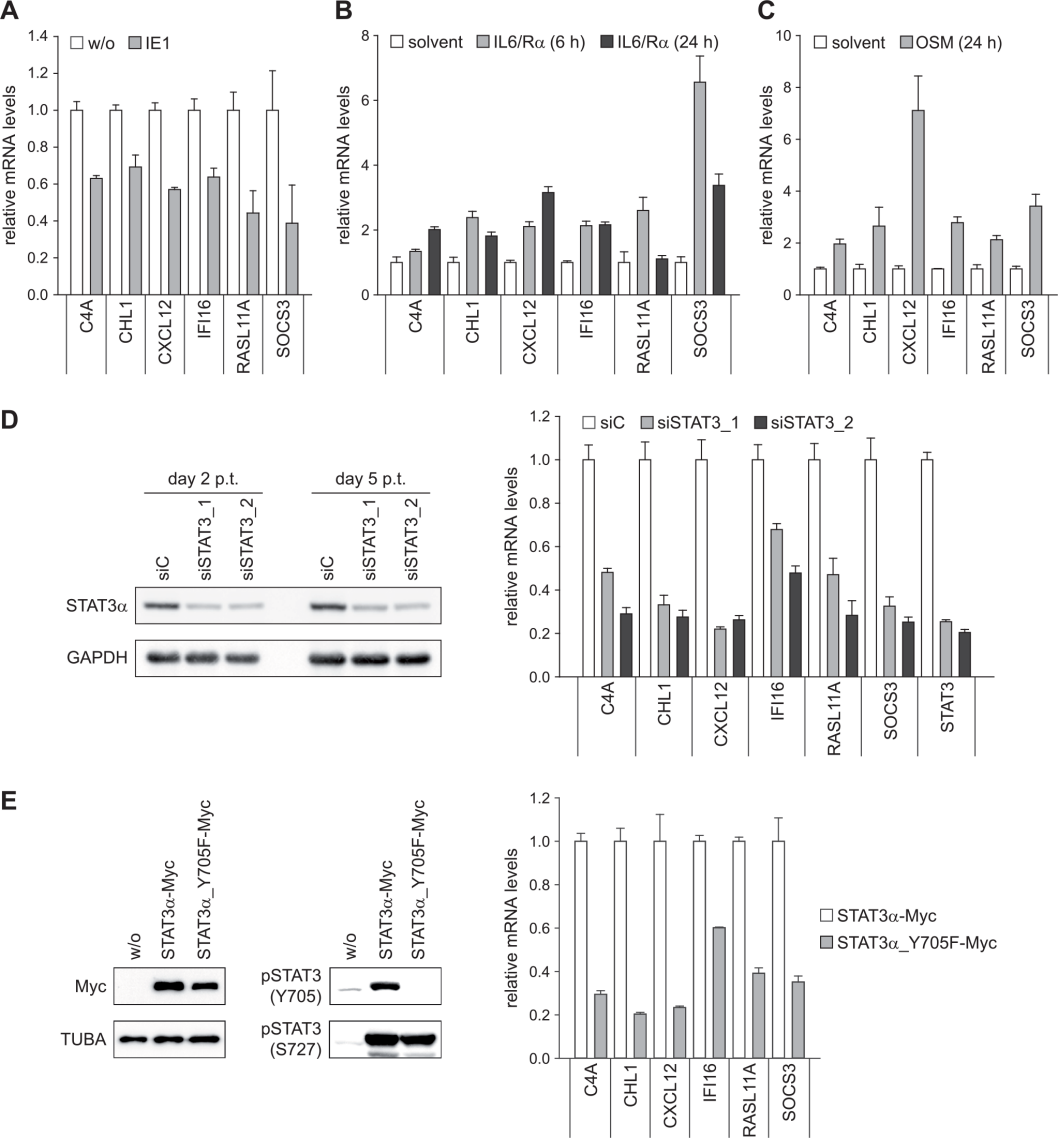
Human genes repressed by IE1 are IL6- and OSM-responsive pSTAT3 target genes. (A) TetR (w/o) and TetR-IE1 (IE1) cells were treated with dox for 72 h. Relative mRNA levels were determined by RT-qPCR with primers specific for the C4A, CHL1, CXCL12, IFI16, RASL11A or SOCS3 genes. Results were normalized to TUBB, and means and standard deviations of biological triplicates are shown in comparison to TetR cells (set to 1). (B) TetR cells were treated with solvent or IL6 plus IL6R (IL6/Rα) for 6 h or 24 h. Relative mRNA levels in comparison to solvent-treated cells (set to 1) were determined by RT-qPCR with primers specific for the indicated genes. Results were normalized to TUBB, and means and standard deviations of biological triplicates are shown. (C) TetR cells were treated with solvent or OSM for 24 h. Relative mRNA levels in comparison to solvent-treated cells (set to 1) were determined by RT-qPCR with primers specific for the indicated genes. Results were normalized to TUBB, and means and standard deviations of biological triplicates are shown. (D) TetR cells were transfected with the indicated siRNA duplexes. Two and five days post transfection (p.t.), whole cell protein extracts were prepared and subjected to immunoblotting for STAT3α and GAPDH (left panel). Five days post transfection, relative mRNA levels were determined by RT-qPCR with primers specific for the indicated genes. Results were normalized to TUBB, and means and standard deviations of two biological and two technical replicates are shown in comparison to control siRNA-transfected cells (set to 1) (right panel). (E) Whole cell protein extracts from TetR cells without (w/o) or with stable expression of the indicated STAT3α-Myc proteins were subjected to immunoblotting for STAT3 (Myc tag), TUBA, pSTAT3 (Y705) and pSTAT3 (S727) (left panel). Total RNA samples from TetR cells overexpressing either wild-type STAT3α-Myc or STAT3α_Y705F-Myc were subjected to RT-qPCR with primers specific for the indicated genes. Results were normalized to TUBB, and means and standard deviations of biological triplicates are shown in comparison to cells expressing wild-type STAT3α-Myc (set to 1) (right panel).

Out of the genes identified to be repressed by IE1, we selected six (C4A, CHL1, CXCL12, IFI16, RASL11A and SOCS3) for validation by reverse transcriptase quantitative PCR (RT-qPCR). The genes were selected to reflect the full range of repression magnitudes and kinetics measured by GeneChip analysis. The RT-qPCR approach confirmed IE1-dependent down-regulation of all tested genes (Fig 2A). Many genes identified to be repressed by IE1, such as CXCL12, IFI16 and SOCS3, are known targets of STAT3 or its upstream activators including IL6 and OSM (Tables 1 and 2). However, to our knowledge, C4A, CHL1 and RASL11A have not been previously linked to activation by STAT3, IL6 or OSM. We therefore examined the effects of IL6 and OSM treatment on the mRNA levels of our select set of IE1-repressed genes. Since fibroblasts do not express sufficient levels of the IL6 receptor α subunit (IL6R) to mount a robust response, IL6 was used in combination with a soluble form of IL6R. The results demonstrate that all tested genes are activated by both IL6 and OSM, although to varying degrees (Fig 2B and 2C). To investigate whether these genes are also STAT3-responsive, STAT3 was silenced with two different siRNA duplexes. Both siRNAs were equally efficient in knocking-down STAT3 expression as confirmed by immunoblotting (Fig 2D, left panel). Following depletion of STAT3 with either of the two siRNAs, all six tested genes exhibited reduced levels of expression (Fig 2D, right panel). Likewise, STAT3 knock-down using two different dox-inducible shRNA constructs demonstrated STAT3-responsive expression for 12 out of 14 tested genes (S1 Fig). Finally, we tested the effects of a mutant STAT3 protein (STAT3α_Y705F), which is expressed to similar levels as the wild-type protein and resistant to Y705 but not S727 phosphorylation (Fig 2E, left panel). This mutant protein is known to act in a trans-dominant negative fashion on expression of STAT3-responsive genes [66]. Accordingly, overexpression of STAT3α_Y705F resulted in reduced levels of all tested genes repressed by IE1 (Fig 2E, right panel).

These findings support the conclusion that most genes found to be down-regulated by IE1 in our system are IL6- and OSM-responsive pSTAT3 target genes, including genes not previously linked to this pathway.

### Physical and functional interaction with STAT3 maps to amino acids 410 to 445 in IE1

**Fig 3:**
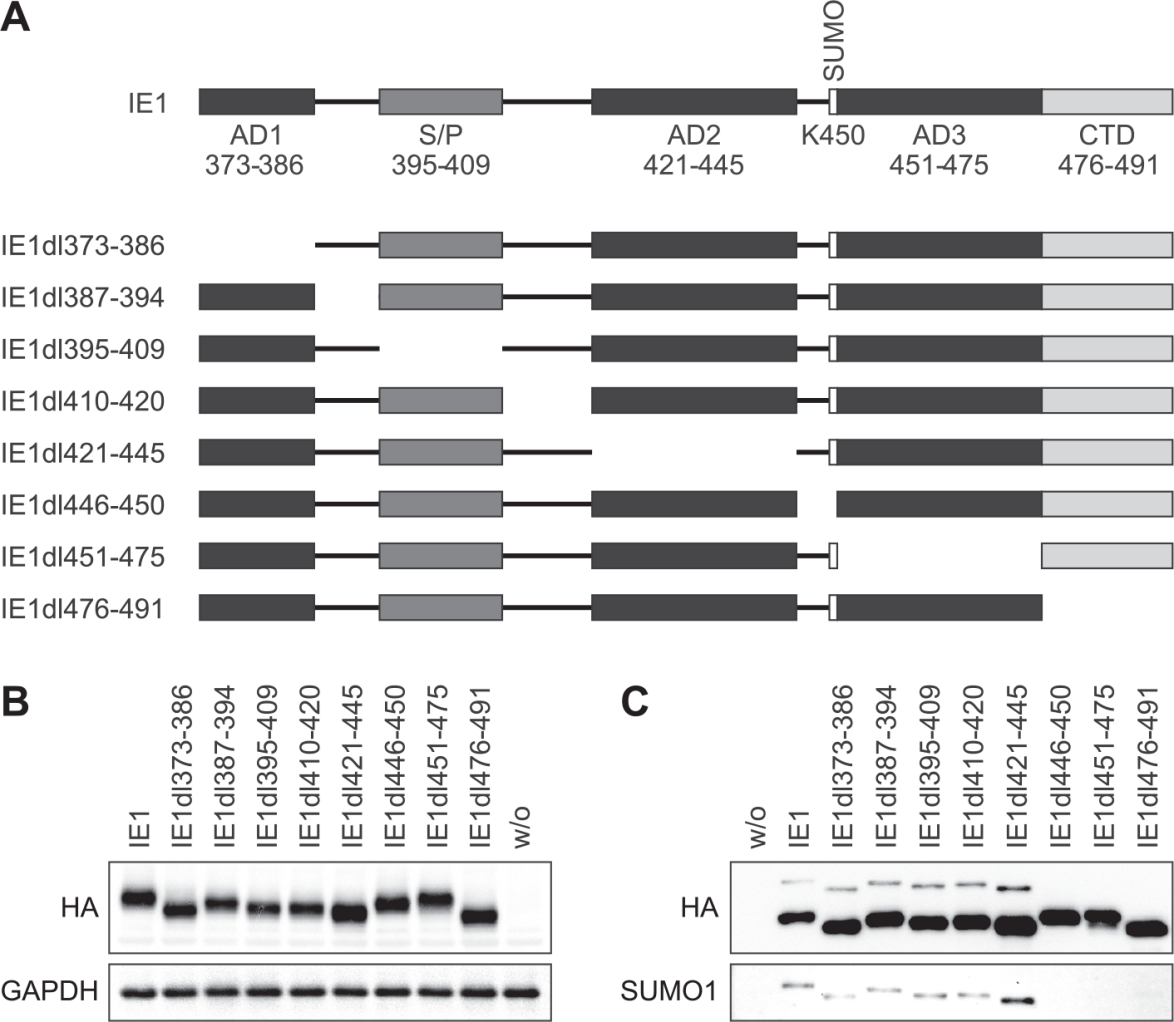
Systematic deletion analysis of C-terminal IE1 residues 373–491. (A) Schematic overview of amino acids 373–491 in the tested wild-type and mutant IE1 proteins. Positions of the low-complexity motifs (acidic domains AD1-3 and serine/proline-rich region S/P), the SUMOylation site (K450) and the chromatin tethering domain (CTD) are shown. (B) TetR cells without (w/o) or with inducible expression of the indicated HA-tagged wild-type or mutant IE1 proteins were treated with dox for 72 h. Whole cell protein extracts were prepared and analyzed by immunoblotting for IE1 (HA tag) and GAPDH. (C) TetR cells without (w/o) or with inducible expression of the indicated HA-tagged wild-type or mutant IE1 proteins were treated with dox for 72 h. Whole cell extracts prepared in the presence of N-ethylmaleimide were used for immunoprecipitation with anti-HA-agarose, and samples were analyzed by immunoblotting for IE1 (HA tag) and SUMO1.

Next, we set out to map the physical determinants of STAT3-directed repression in IE1 and to relate them to other known activities of the viral protein. Previous work by us and Huh *et al*. has narrowed down STAT2 interaction to a region between amino acids 373 and 445 or 421 and 475, respectively, in the C-terminal quarter of IE1 [53, 54] suggesting that STAT3 might also bind to this part of the viral protein. The four low complexity stretches (AD1, S/P, AD2 and AD3), the sequences (including the SUMOylation motif) linking these stretches and the CTD were individually deleted resulting in a set of eight mutant proteins spanning the entire IE1 C-terminal region between amino acids 373 and 491 (Fig 3A). Subsequently, MRC-5 cells were transduced with lentiviruses expressing the wild-type or mutant IE1 proteins to generate a correspondent set of dox-inducible cell lines. Following induction, the steady-state IE1 levels in each mutant cell line were comparable to those of TetR-IE1 cells expressing the full-length viral protein (Fig 3B). The IE1 mutants lacking an intact VKSE motif (IE1dl446-450 and dl451-475) failed to undergo SUMOylation, as expected. IE1dl476-491 was not SUMOylated either, suggesting that the CTD is required in addition to the VKSE motif for this posttranslational modification (Fig 3C).

**Fig 4:**
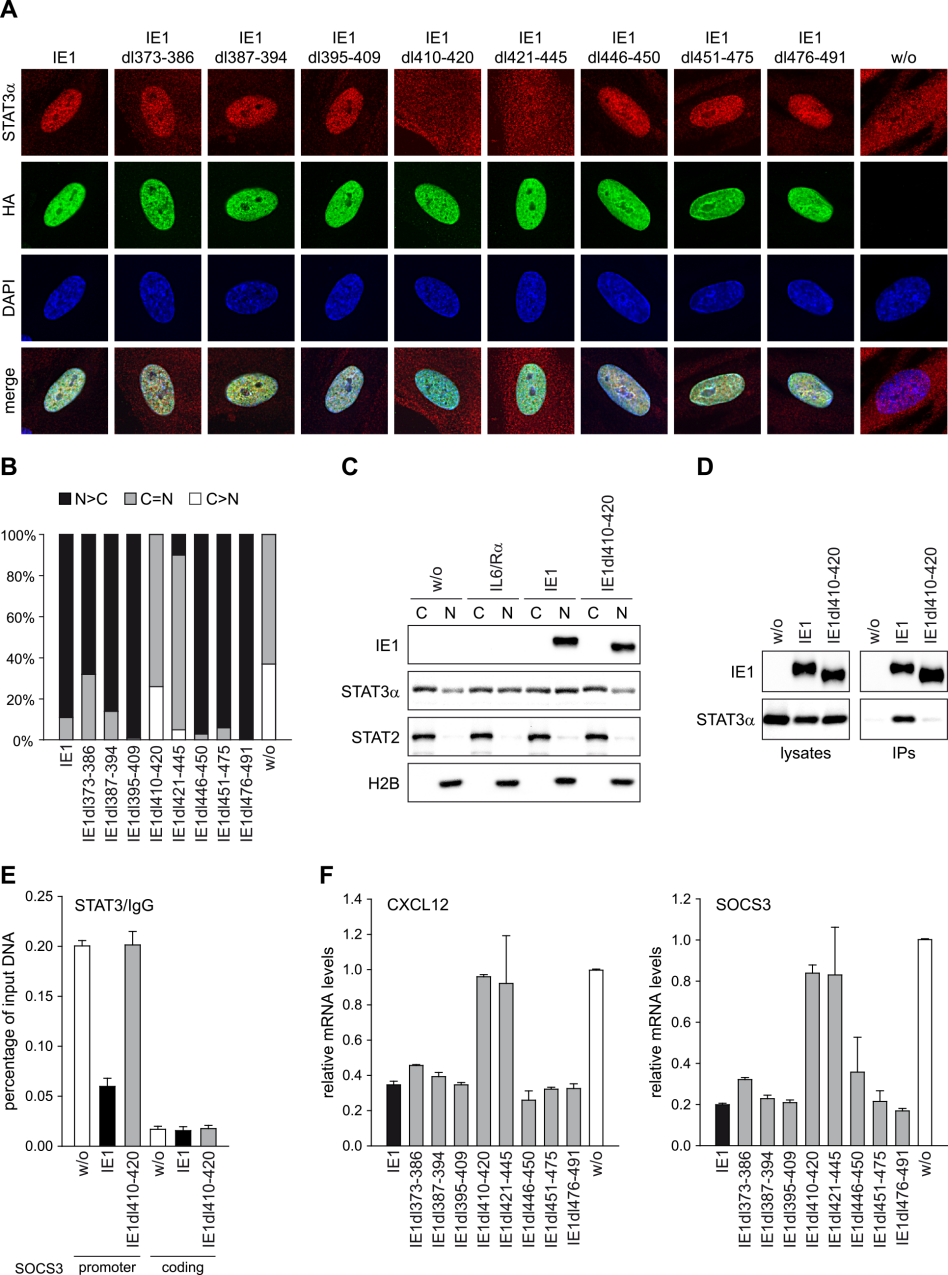
Residues within IE1 region 410–445 are required for targeting of STAT3 and down-regulation of STAT3-responsive genes. (A) TetR cells without (w/o) or with inducible expression of the indicated HA-IE1 proteins were treated with dox for 48 h. During the final 24 h of dox treatment, cells were kept in medium with 0.5% FBS. Subcellular localization of endogenous STAT3α in IE1 expressing cells was analyzed by indirect immunofluorescence microscopy. Samples were simultaneously reacted with a rabbit monoclonal antibody to STAT3α and a mouse monoclonal antibody to HA-tagged IE1, followed by incubation with a rabbit-specific Alexa Fluor 594 conjugate and a mouse-specific Alexa Fluor 488 conjugate. Host cell nuclei were visualized by 4',6-diamidino-2-phenylindole (DAPI) staining. Additionally, merge images of STAT3α, IE1 and DAPI signals are presented. (B) The percentage of cells with i) predominantly nuclear STAT3α staining (N>C), ii) equally strong nuclear and cytoplasmic STAT3α staining (N = C) and iii) predominantly cytoplasmic STAT3α staining (C>N) was determined for 100 randomly selected cells per sample described in (A). (C) TetR cells without or with inducible expression of HA-tagged wild-type IE1 or IE1dl410-420 were treated with dox for 72 h and with solvent (w/o) or IL6 plus IL6R (IL6/Rα) for 30 min. Cytoplasmic and nuclear extracts were prepared and analyzed by immunoblotting for histone H2B, STAT2, STAT3α and IE1. (D) TetR cells without (w/o) or with inducible expression of HA-tagged wild-type IE1 or IE1dl410-420 were treated with dox for 72 h. Whole cell extracts were prepared and used for immunoprecipitations (IPs) with anti-HA-agarose. Samples of lysates and immunoprecipitates were analyzed by immunoblotting for IE1 and STAT3α. (E) TetR cells without (w/o) or with inducible expression of HA-tagged wild-type IE1 or IE1dl410-420 were treated with dox for 72 h and with IL6 plus IL6R for 30 min. Samples were subjected to ChIP with rabbit polyclonal antibodies to STAT3 or normal rabbit IgG and primers specific for sequences in the SOCS3 promoter or coding region. The percentage of output versus input DNA is presented as the difference between STAT3 and normal IgG ChIPs. Means and standard deviations of two biological and two technical replicates are shown. (F) TetR cells without (w/o) or with inducible expression of the indicated HA-tagged wild-type or mutant IE1 proteins were treated with dox for 72 h. Relative mRNA expression levels were determined by RT-qPCR with primers specific for the STAT3 target genes CXCL12 and SOCS3. Results were normalized to TUBB, and means and standard deviations of two biological and two technical replicates are shown in comparison to IE1-negative TetR cells (set to 1).

All mutant proteins displayed nuclear localization undistinguishable from wild-type IE1, as determined by immunofluorescence microscopy (Fig 4A). When testing for nuclear accumulation of STAT3, cells expressing IE1dl373-386, dl387-394, dl395-409, dl446-450, dl451-475 or dl476-491 closely resembled wild-type expressing TetR-IE1 cells in exhibiting predominantly nuclear diffuse STAT3 staining (>60% of cells) or, less frequently, a balanced distribution of STAT3 between the nucleus and cytoplasm (<40% of cells). By contrast, in most cells expressing IE1dl410-420, dl421-445 or no IE1, STAT3 was either evenly distributed across the nucleus and cytoplasm (>60% of cells) or predominantly present in the cytoplasm (<40% of cells), but rarely if at all enriched in the nucleus (Fig 4A and 4B). The immunofluorescence results were independently confirmed by subcellular fractionation analysis showing reduced STAT3 (but not STAT2) nuclear accumulation in cells expressing IE1dl410-420 or no IE1 compared to wild-type IE1 expressing or IL6-treated cells (Fig 4C). In agreement with the subcellular localization analyses, IE1 amino acids 405 to 491 were sufficient (S2 Fig) and amino acids 410 to 420 were required (Fig 4D) for physical interaction with STAT3 when mutants were compared to the full-length viral protein in co-immunoprecipitation assays (Fig 4D). Unlike wild-type IE1, the mutant protein also proved to be unable to interfere with STAT3 binding to SOCS3 promoter sequences, as analyzed by chromatin immunoprecipitation (ChIP) assay (Fig 4E). Finally, repressed expression of the STAT3-responsive CXCL12 and SOCS3 genes was observed with the wild-type and all tested mutant proteins except for IE1dl410-420 and dl421-445 (Fig 4F).

Collectively, these results indicate that the STAT3-related activities of IE1 do not involve SUMOylation or nucleosome binding. Instead, residues in a region of IE1 that links the S/P and AD2 low complexity stretches (amino acids 410 to 420) as well as residues within the AD2 motif (amino acids 421–445) are required for interaction with STAT3 and subsequent repression of STAT3-responsive genes.

### Inhibition of STAT3-dependent and activation of STAT1-dependent signaling by IE1 co-segregate

**Fig 5:**
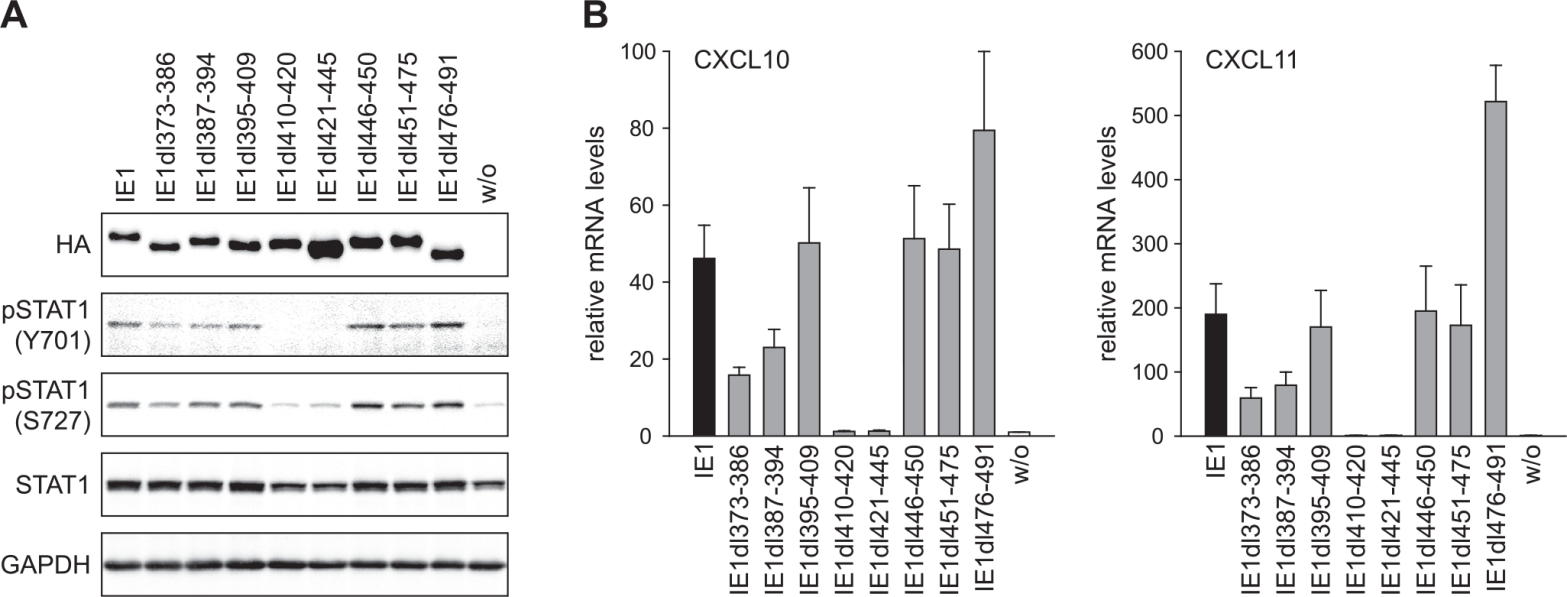
Residues within IE1 region 410–445 are required for phosphorylation of STAT1 and up-regulation of IFNγ-responsive genes. (A) TetR cells without (w/o) or with inducible expression of the indicated HA-tagged wild-type or mutant IE1 proteins were treated with dox for 72 h. Whole cell protein extracts were prepared and analyzed by immunoblotting for IE1 (HA tag), pSTAT1 (Y701), pSTAT1 (S727), total STAT1 and GAPDH. (B) TetR cells without (w/o) or with inducible expression of the indicated HA-tagged wild-type or mutant IE1 proteins were treated with dox for 72 h. Relative mRNA expression levels were determined by RT-qPCR with primers specific for the STAT1 target genes CXCL10 and CXCL11. Results were normalized to TUBB, and means and standard deviations of two biological and two technical replicates are shown in comparison to IE1-negative TetR cells (set to 1).

Our previous work has demonstrated that IE1 triggers a type II IFN-like response via a mechanism that is dependent on tyrosine (Y701) phosphorylation and enhanced by serine (S727) phosphorylation of STAT1 induced by the viral protein [21]. To investigate whether the STAT1- and STAT3-related activities of IE1 are linked, we subjected our wild-type and mutant IE1 expressing cell lines to immunoblotting for Y701- and S727-phosphorylated STAT1. Again, all but the IE1dl410-420 and dl421-445 mutants induced STAT1 Y701 and S727 phosphorylation in a fashion comparable to the wild-type viral protein (Fig 5A). Cells expressing these two mutants also exhibited lower overall STAT1 levels compared to cells expressing the wild-type or other mutant proteins, most likely because pSTAT1 positively regulates its own expression. Likewise, when tested for induction of genes representative of pSTAT1-responsive type II ISGs (CXCL10 and CXCL11) by RT-qPCR, the IE1dl410-420 and dl421-445 proteins were severely defective. All other mutants were active for CXCL10 and CXCL11 induction, although IE1dl373-386 and dl387-394 displayed reduced and IE1dl476-491 protein increased activities compared to the wild-type (Fig 5B). We suspect that mutations between amino acids 373 and 394 may indirectly affect IE1-STAT3 complex formation, perhaps by impacting the viral protein’s overall structure. Conversely, increased nucleoplasmic localization may explain why the CTD-deficient IE1 mutant (IE1dl410-420) is more potent than the chromatin-associated viral protein. We also observed that the IE1-dependent repression of genes responsive to STAT3, IL6 or/and OSM tends to precede the activation of genes responsive to STAT1 or/and IFNγ by IE1 (S3 Fig).

These results suggest that the effects IE1 exerts on the STAT1- and STAT3-dependent signaling pathways are related and indicate that the former might depend on the latter.

### STAT3 depletion recapitulates and IL6ST or JAK1 depletion disrupts IE1-induced expression of STAT1-responsive genes

**Fig 6:**
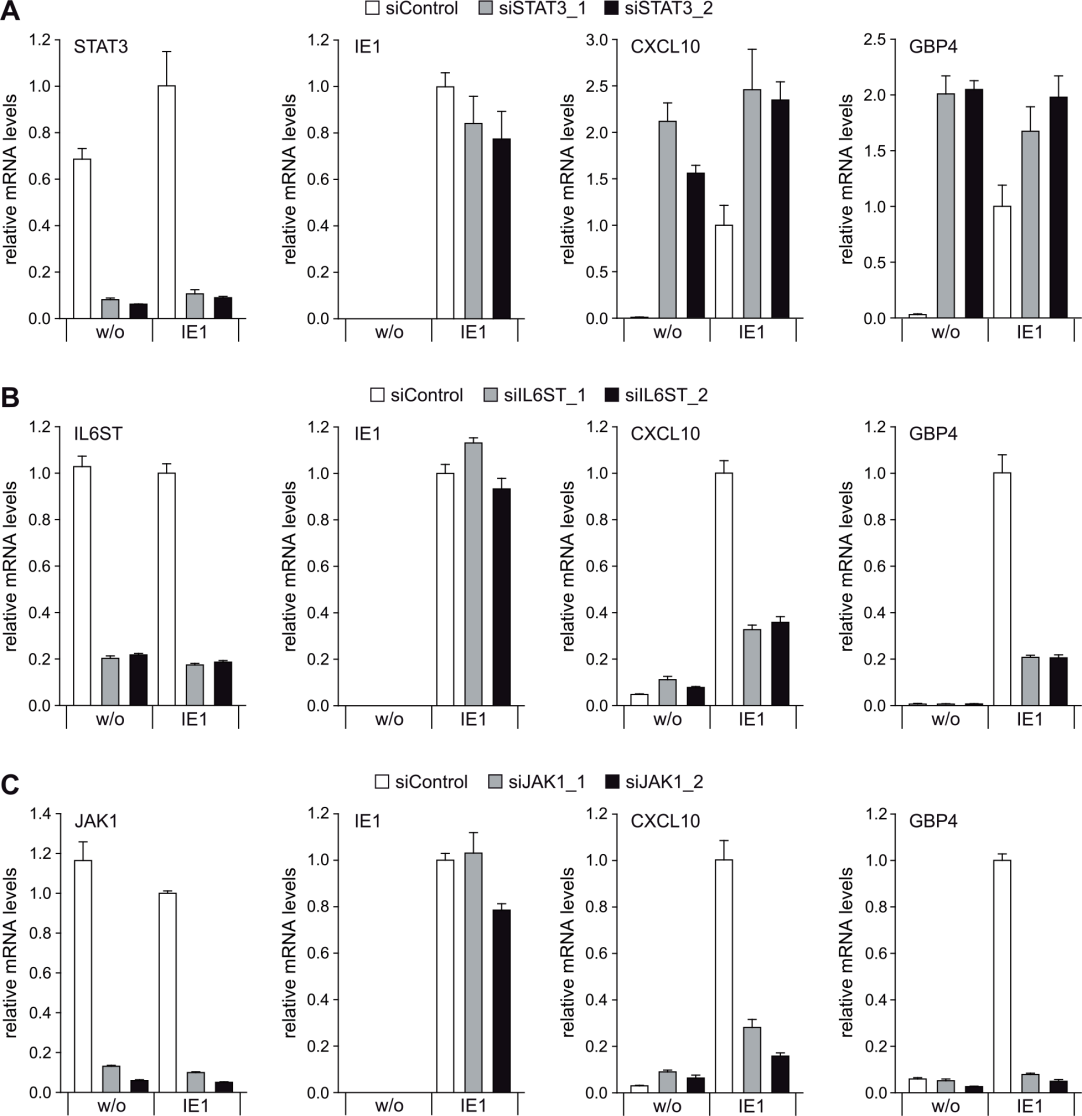
Knock-down of STAT3 recapitulates, while knock-down of IL6ST or JAK1 disrupts IE1-mediated induction of IFNγ-stimulated genes. (A) TetR (w/o) or TetR-IE1 (IE1) cells were transfected with a control siRNA or two different siRNAs specific for STAT3. From 48 h post siRNA transfection, cells were treated with dox for 72 h. Relative mRNA levels were determined by RT-qPCR for STAT3, IE1 and the STAT1 target genes CXCL10 and GBP4. Results were normalized to TUBB, and means and standard deviations of two biological and two technical replicates are shown in comparison to control siRNA-transfected TetR-IE1 cells (set to 1). (B) TetR (w/o) or TetR-IE1 (IE1) cells were transfected with a control siRNA or two different siRNAs specific for IL6ST. From 48 h post siRNA transfection, cells were treated with dox for 72 h. Relative mRNA levels were determined by RT-qPCR for IL6ST, IE1, CXCL10 and GBP4. Results were normalized to TUBB, and means and standard deviations of two biological and two technical replicates are shown in comparison to control siRNA-transfected TetR-IE1 cells (set to 1). (C). TetR (w/o) or TetR-IE1 (IE1) cells were transfected with a control siRNA or two different siRNAs specific for JAK1. From 48 h post siRNA transfection, cells were treated with dox for 72 h. Relative mRNA levels were determined by RT-qPCR for JAK1, IE1, CXCL10 and GBP4. Results were normalized to TUBB, and means and standard deviations of two biological and two technical replicates are shown in comparison to control siRNA-transfected TetR-IE1 cells (set to 1).

In the next step, we addressed the mechanism underlying the proposed link between the IE1-related effects on STAT3- and STAT1-dependent signaling. To this end, we used two siRNA duplexes each to individually knock-down expression from three essential genes (STAT3, IL6ST and JAK1) of the IL6-type pSTAT3 signaling pathway and examined the consequences on IE1-mediated induction of pSTAT1-responsive type II ISGs (CXCL10 and GBP4). Either of the target-specific siRNAs reduced the levels of the corresponding mRNA by ≥80%, compared to a non-specific siRNA, without significantly affecting the IE1 mRNA levels (Fig 6A–6C). Notably, STAT3 knock-down by either of the two specific siRNAs lead to a dramatic increase in CXCL10 and GBP4 transcript levels (in the absence of IE1) and enhanced IE1-dependent ISG induction. Conversely, IL6ST or JAK1 knock-down had little effect on CXCL10 and GBP4 transcript levels, but markedly reduced ISG induction by IE1 (Fig 6A–6C). In comparison, IFNGR1 knock-down affected ISG (CXCL9, CXCL10 and CXCL11) activation by IE1 only slightly (S4 Fig) confirming that this effect is largely independent from upstream components of the type II IFN signaling pathway including IFNγ [21].

These results indicate that the pSTAT1-dependent IFNγ-like transcriptional response observed in the presence of IE1 depends on upstream components of the IL6-type (but not IFNγ-type) signaling pathway and may involve targeting of STAT3.

### IE1 rewires an upstream IL6-type to a downstream IFNγ-like response

**Fig 7:**
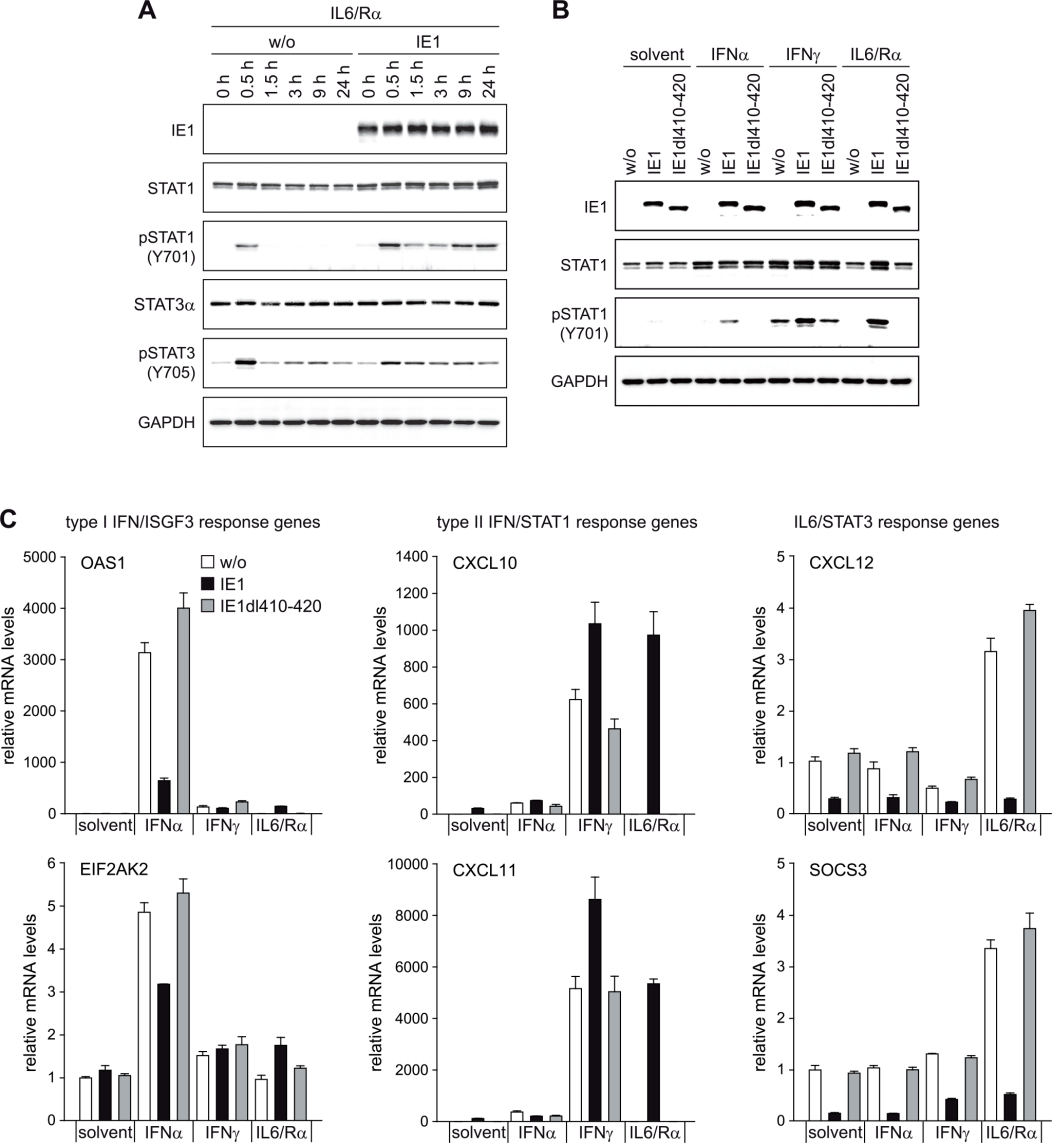
IE1 switches an IL6-type to an IFNγ-like response. (A) TetR (w/o) or TetR-IE1 (IE1) cells were treated with dox for 72 h and with IL6 plus IL6R (IL6/Rα) for the indicated times. Whole cell protein extracts were prepared and analyzed by immunoblotting for IE1, total STAT1, pSTAT1 (Y701), total STAT3α, pSTAT3 (Y705) and GAPDH. (B) TetR (w/o) or TetR-IE1 cells expressing HA-tagged wild-type IE1 or IE1dl410-420 were treated with dox for 72 h and with solvent, IFNα, IFNγ or IL6 plus IL6R (IL6/Rα) for 24 h. Whole cell protein extracts were analyzed by immunoblotting for IE1, total STAT1, pSTAT1 (Y701) and GAPDH. (C) TetR (w/o) or TetR-IE1 cells expressing HA-tagged wild-type IE1 or IE1dl410-420 were treated with dox for 72 h and with solvent, IFNα, IFNγ or IL6 plus IL6R (IL6/Rα) for 24 h. Relative mRNA levels were determined by RT-qPCR for the type I IFN/STAT2 target genes OAS1 and EIF2AK2 (protein kinase R) (left panels), the type II IFN/STAT1 target genes CXCL10 and CXCL11 (middle panels) and the IL6/STAT3 target genes CXCL12 and SOCS3 (right panels). Results were normalized to TUBB, and means and standard deviations of biological triplicates are shown in comparison to solvent-treated TetR cells (set to 1).

Based on the above results, we hypothesized that IE1 may redirect IL6-related signaling away from STAT3 and towards STAT1 activation. In accordance with this hypothesis, IL6 triggered robust and prolonged STAT1 Y701 phosphorylation in the presence of wild-type IE1, but not in the presence of IE1dl410-420 or in the absence of the viral protein (Fig 7A and 7B). In fact, the combination of IL6 and IE1 was at least equally efficient in mediating STAT1 activation as IFNγ. Consistently, IL6 also caused ~1,000-fold increased expression of pSTAT1-responsive type II ISGs (CXCL10 and CXCL11) in cells with wild-type IE1 compared to cells with IE1dl410-420 or no viral protein (Fig 7C). We further observed that IFNγ was more effective in inducing STAT1 Y701 phosphorylation and type II ISG induction when wild-type IE1 was expressed. Instead, wild-type IE1 inhibited induction of type I ISGs by IFNα in accordance with previous reports [52–54] (Fig 7B and 7C). Finally, wild-type IE1 but not IE1dl410-420 inhibited both basal and cytokine-induced expression of IL6/STAT3-responsive genes (CXCL12 and SOCS3) (Fig 7C).

From these results we conclude that IE1 disconnects upstream IL6-type from downstream STAT3-dependent signaling, reconnecting it to downstream STAT1 signaling and related gene expression.

### hCMV infection rewires IL6-type to IFNγ-like signaling in an IE1-dependent manner

**Fig 8:**
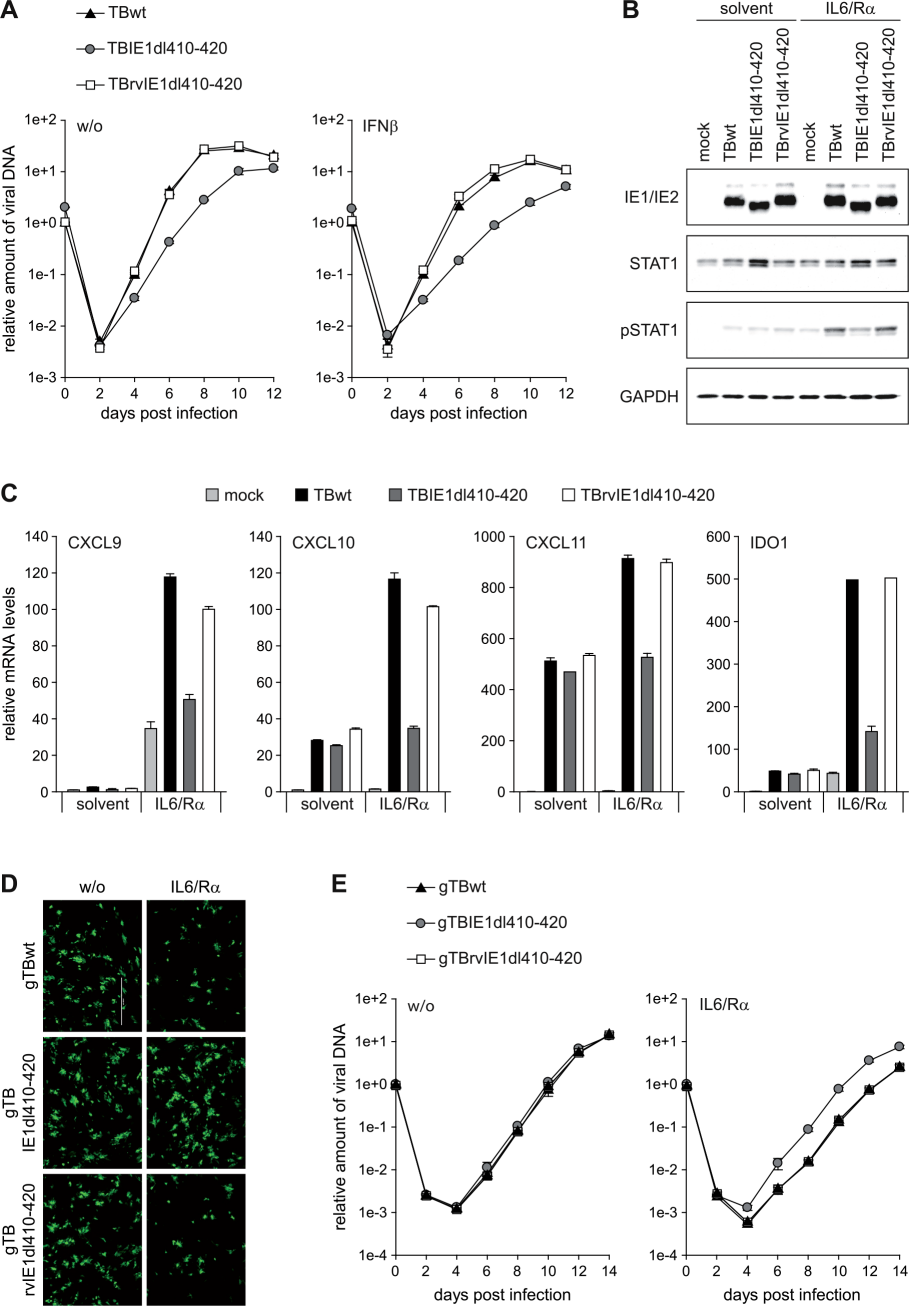
IE1 rewires IL6 signaling to STAT1 activation during hCMV infection. (A) MRC-5 cells were infected with TBwt, TBIE1dl410-420 or TBrvIE1dl410-420 at low input multiplicity (0.1 PFU/cell) in the absence (w/o) or presence of exogenous IFNβ. Culture media were replaced every 24 h, and viral replication was assessed by qPCR-based relative quantification of hCMV DNA from culture supernatants at the indicated times post infection with primers specific for the viral UL86 sequence. Data are presented as means and standard deviations from three independent infections. (B) MRC-5 cells were mock-infected or infected with TBwt, TBIE1dl410-420 or TBrvIE1dl410-420 at a high input multiplicity (5 PFU/cell). At 6 h post infection, cultures were treated with solvent or IL6 plus IL6R (IL6/Rα). At 24 h post infection, whole cell protein extracts were prepared and analyzed by immunoblotting for IE1/IE2, total STAT1, pSTAT1 (Y701) and GAPDH. (C) MRC-5 cells were mock-infected or infected with TBwt, TBIE1dl410-420 or TBrvIE1dl410-420 at a high input multiplicity (5 PFU/cell). At 6 h post infection, cultures were treated with solvent or IL6 plus IL6R (IL6/Rα). At 24 h post infection, relative mRNA levels were determined by RT-qPCR for the STAT1 target genes CXCL9, CXCL10, CXCL11 and IDO. Results were normalized to TUBB, and means and standard deviations of biological triplicates are shown in comparison to solvent-treated mock-infected cells (set to 1). (D) STAT2-deficient human skin fibroblasts were infected with gTBwt, gTBIE1dl410-420 or gTBrvIE1dl410-420 at low input multiplicity (0.1 PFU/cell) in the absence (w/o) or presence of IL6 plus IL6Rα (IL6/Rα). Every 48 h, half of the culture media was replaced and viral replication was assessed at day 6 post infection by fluorescence microscopy. (E) STAT2-deficient human skin fibroblasts were infected with gTBwt, gTBIE1dl410-420 or gTBrvIE1dl410-420 at low input multiplicity (0.1 PFU/cell) in the absence (w/o) or presence of IL6 plus IL6Rα (IL6/Rα). Every 48 h, half of the culture media was replaced and viral replication was assessed by qPCR-based relative quantification of hCMV DNA from culture supernatants with primers specific for the viral UL86 sequence. Data are presented as means and standard deviations from two biological and two technical replicates.

To be able to test whether the IE1-STAT3 interaction diverts IL6 signaling also in the context of infection, we derived a mutant virus (TBIE1dl410-420) specifically lacking the sequence encoding IE1 amino acids 410 to 420 from a bacterial artificial chromosome (BAC) clone of the hCMV TB40/E strain. In addition, a virus in which the mutated sequence was reverted to wild-type (TBrvIE1dl410-420) was constructed. The integrity and identity of the BACs underlying the mutant and revertant viruses were verified in comparison to the wild-type (TBwt) by restriction fragment and PCR analysis, respectively (S5 Fig). Following infection of MRC-5 cells, an increase in nuclear localization of STAT3 compared to mock-infected cells was observed with the TBwt and TBrvIE1dl410-420 but not the TBIE1dl410-420 virus (S6 Fig). At low input multiplicity, the TBIE1dl410-420 virus exhibited attenuated replication and increased sensitivity to exogenous IFNβ compared to the TBwt and TBrvIE1dl410-420 viruses (Fig 8A). At high multiplicity of infection, the IE1dl410-420 protein accumulated to similar levels as the full-length protein expressed from TBwt or TBrvIE1dl410-420 (Fig 8B). However, significant IL6-dependent STAT1 Y701 phosphorylation was only observed with the wild-type and revertant but not the mutant virus at both early (24 h) and late times (72 h) post infection (Fig 8B and S7A Fig). Upon TBwt infection, several type II ISGs (CXCL9, CXCL10, CXCL11 and IDO) exhibited varying degrees of activation at 24 h post infection in accordance with previous reports [21, 67–76]. Following addition of IL6 to the infected cells, expression of the four tested ISGs increased by ~2- to >30-fold. This increase was significantly stronger in TBwt and TBrvIE1dl410-420 compared to TBIE1dl410-420 infections (Fig 8C and S7B Fig).

These results indicate that the IE1-STAT3 interaction is not only sufficient to rewire IL6-type to IFNγ-like signaling, but also largely required to mediate this response during hCMV infection.

### Rewired IL6-type to IFNγ-like signaling tempers hCMV replication

The phenotype of the TBdlIE1410-420 mutant virus (Fig 8A) suggests that the diverted signaling linked to IE1-STAT3 interaction may be beneficial to hCMV replication. However, STAT3 knock-down turned out to inhibit replication of both TBwt and TBdlIE1410-420 (S8 Fig). Moreover, STAT2 binding maps to a region overlapping IE1 amino acids 410–420 [53] and TBdlIE1410-420 is hypersensitive to IFNβ (Fig 8A). Thus, the replication defect of TBdlIE1410-420 may derive from either or both inhibition of type I IFN signaling (via STAT2 binding) or rewiring of IL6/IFNγ signaling (via STAT3 binding). To discriminate between the consequences of STAT2 and STAT3 targeting by IE1, infections with EGFP expressing hCMV strains were performed in human fibroblasts lacking any STAT2 protein. In the absence of STAT2, replication of the gTBIE1dl410-420 mutant did not differ from the wild-type (gTBwt) and revertant (gTBrvIE1dl410-420) virus. Furthermore, addition of IL6 selectively reduced the replication efficiency of gTBwt and gTBrvIE1dl410-420 but not gTBIE1dl410-420 (Fig 8D and 8E).

These results indicate that the type II IFN-like response mediated by IL6-type cytokines in the presence of IE1 moderates rather than promotes hCMV replication, revealing an unanticipated temperance activity encoded in the viral protein.

## Discussion

Decades of research have established that hCMV IE1 is a promiscuous transcriptional activator of viral and cellular genes [42, 65]. In contrast, evidence supporting transcriptional repression by IE1 is much scarcer. Our study reveals that IE1 is as much a repressor as it is an activator of host gene expression. This finding is unexpected and challenges the view that the viral protein is essentially a transcriptional activator. Many of the genes found to be down-regulated by IE1 have been previously shown to be up-regulated by pSTAT3 or/and upstream regulators in this pathway including cytokines of the IL6 family. Beyond that, we identified several new pSTAT3 targets among the genes that are subject to IE1 repression. We predict that many more of the genes repressed by IE1 than those listed in Table 1 and marked as STAT3-responsive in Table 2 will turn out to be activated by signaling via pSTAT3. Although, to our knowledge, this is the first systematic genome-wide analysis of transcriptional repression by IE1, our results are consistent with earlier findings linking the viral protein to down-regulation of the genes encoding glial fibrillary acidic protein [77, 78] and SOCS3 [30], both of which are activated by pSTAT3 [79–82]. Our results are also in line with previous reports on inhibition of IL6 signaling by hCMV, although hCMV-dependent activation of IL6 signaling has also been observed [30, 83]. The possibility that IL6 signaling is induced and subsequently blocked in cells hit by infectious virus (expressing IE1), but induced without being blocked in bystanding cells hit by non-infectious virus (not expressing IE1) may reconcile these seemingly conflicting findings.

Our conclusions regarding the mechanism underlying JAK-STAT-related transcriptional repression and activation by IE1 result partly from the analysis of mutant proteins. These analyses map all activities linked to repression of pSTAT3- and activation of pSTAT1-responsive genes, including IE1-STAT3 complex formation, to a region between amino acids 410 and 445 in the C-terminal domain of the viral protein. This region contains residues upstream of and including AD2, a low complexity motif rich in glutamic and aspartic acid. Other interactions mapped to the C-terminal domain of IE1 include binding to chromatin/nucleosomes via the CTD (amino acids 476–491) [59, 61, 62], conjugation to SUMO1 via lysine 450 [57, 58, 84] and binding to STAT2 [52–54]. The CTD proved to be necessary for SUMO1 conjugation, confirming results from previous reports [85, 86] and suggesting that nucleosome binding may be a prerequisite for SUMOylation of IE1. However, nucleosome interaction and SUMOylation were not required for the observed effects IE1 exerts on JAK-STAT signaling. In fact, SUMOylation has been previously shown to negatively regulate the IE1-STAT2 interaction [54]. The relationship between the IE1-STAT2 and IE1-STAT3 interactions is discussed below.

**Fig 9:**
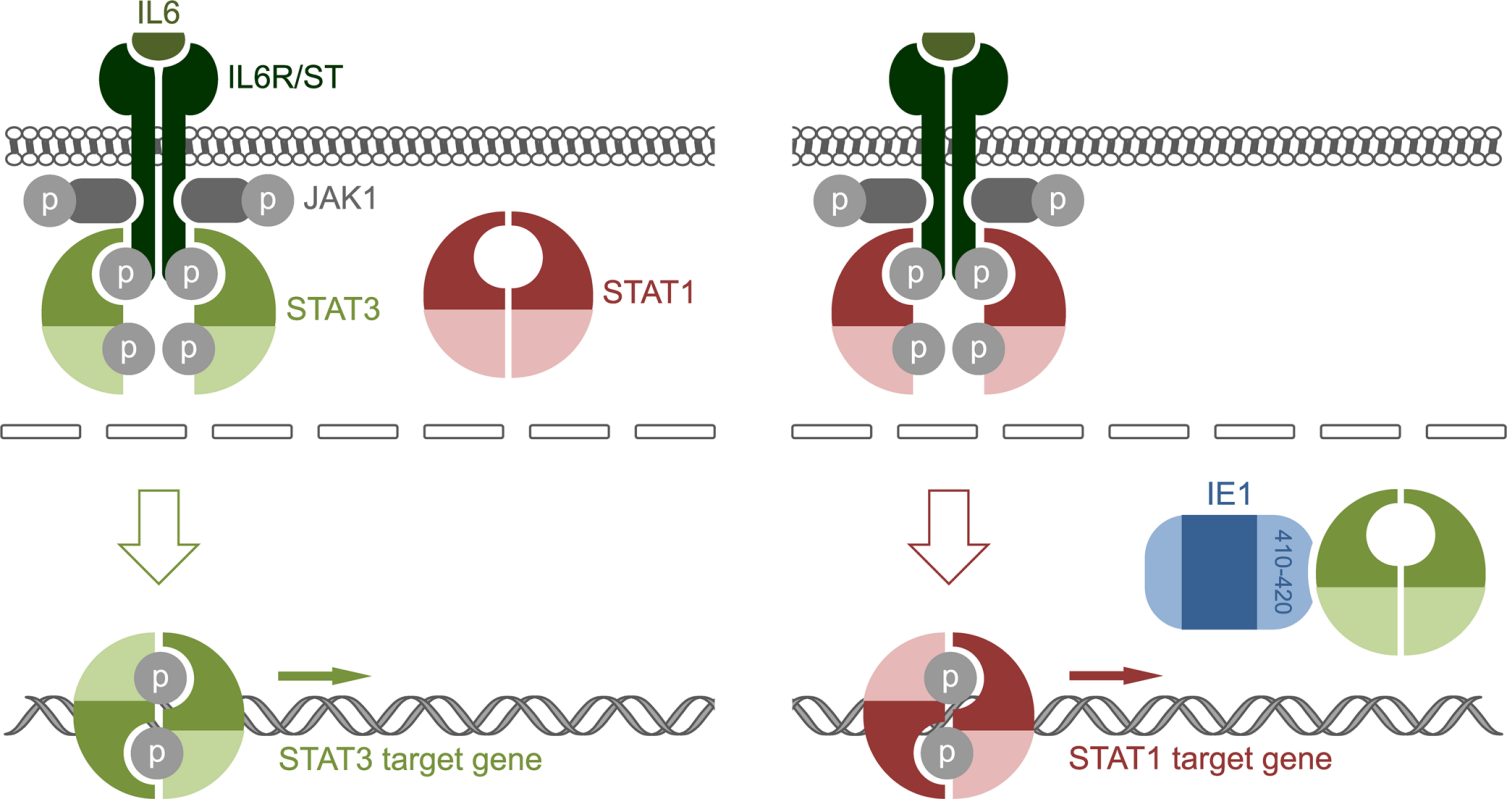
Model linking repression of STAT3-responsive to activation of STAT1-responsive genes by IE1. (Left panel) Binding of IL6 family cytokines (or growth factors) to the extracellular IL6R subunits leads to tyrosine phosphorylation of JAK1 (and other JAK family kinases) associated with the IL6ST receptor subunits. In turn, JAK1 phosphorylates tyrosine residues in IL6ST creating binding sites for STAT3. Following tyrosine phosphorylation by JAK1, pSTAT3 forms active dimers capable of binding DNA. These STAT3 dimers accumulate in the nucleus and activate target gene transcription. STAT1 remains mostly unphosphorylated, cytoplasmic and excluded from binding to the receptor in the absence of IE1. (Right panel) In the presence of IE1, cytoplasmic STAT3 pools become depleted due to formation of a nuclear IE1-STAT3 complex. In the absence of cytoplasmic STAT3, STAT1 binds to the activated IL6ST and undergoes JAK1-mediated tyrosine phosphorylation resulting in active dimers. These STAT1 dimers accumulate in the nucleus, bind to DNA and activate target gene expression resulting in an IFNγ-like response triggered by IL6-type cytokines.

Most previous work has suggested that IE1 activates gene expression through various chromatin-directed mechanisms including recruitment of transcription factors [87–89], inhibition of transcription repressors [90–92], acetylation of histones [93] or reorganization of nucleosomes [94]. Our present study demonstrates that IE1 also passively modulates transcription upstream of chromatin by linking repression of STAT3- to activation of STAT1-responsive human genes. Our results suggest a model (Fig 9) where STAT3, which shuttles through the nucleus independent of phosphorylation [5], forms a nuclear resident complex with IE1. The nuclear retention imposed on STAT3 precludes the protein from being Y705-phosphorylated by its cytoplasmic kinase JAK1. Thus, the STAT3 accumulating in the nucleus in the presence of IE1 is mostly unphosphorylated [30] and therefore unable to bind to and activate pSTAT3-dependent target genes. It remains to be investigated whether IE1 also prevents DNA binding of Y705-phosphorylated STAT3. In the context of activated IL6-type signaling, the nuclear IE1-STAT3 interaction becomes evident as transcriptional repression of pSTAT3-responsive genes. In addition to mediating this repression, the IE1-STAT3 interaction also triggers transcriptional activation. We propose that, in the absence of cytoplasmic STAT3, JAK1 mediates Y701 phosphorylation of STAT1 leading to nuclear accumulation of pSTAT1 and activation of type II ISGs. Type II ISG activation by IE1 has been observed before [21], but seems to contradict a recent report showing that the viral protein disrupts type II IFN signaling by an unknown mechanism [95]. It appears that this mechanism involves indirect consequences of IE1-STAT2 rather than direct IE1-STAT1 binding, just as the activation of type II ISGs is an indirect consequence of the IE1-STAT3 interaction. In fact, direct binding between IE1 and STAT1 has not been demonstrated, although the two proteins may indirectly associate via STAT heterodimer formation [52, 63]. In any case, our model is fully consistent with the fact that depletion of STAT3 phenocopies the IE1-related effects we observe, and that these effects are largely dependent on pSTAT1 [21], IL6ST and JAK1 but not IFNGR1. It is also in line with the observation that IE1 promotes STAT1 phosphorylation and nuclear accumulation [21]. Finally, the model fits the idea that IFNγ‐like responses depend on pSTAT1, but not necessarily on signaling through the cognate receptor. In fact, activation of STAT1 through foreign chimeric receptors proved to be almost as effective in mediating major aspects of an IFNγ response in human cells as activation through the natural receptor [96]. It has also been shown that phosphotyrosine motifs in both IFNGR and IL6ST can serve as docking sites for the STAT1 SH2 domain [97–100]. Two of the four phosphotyrosine motifs in IL6ST are specific for STAT3 while two can recruit both STAT3 and STAT1 with similar affinities [100]. The absence of STAT3 may release competition for the common docking sites, favoring recruitment and activation of STAT1. In accordance with this idea, IL6 triggered an IFNγ-like response including prolonged activation of STAT1 and induction of multiple type II ISGs in mouse cells lacking STAT3 [15]. A similar type II ISG response to STAT3 depletion was observed in human cells (this work). The STAT3/STAT1 cross-regulation seems to result in an integrated signal that can be fine-tuned depending on the cellular context, strongly arguing for flexible rather than fixed wiring of these pathways [14, 16, 17, 101–103].

We anticipate that future work will recognize at least a subset of genes repressed or activated by IE1 as important factors in hCMV infection. In fact, several targets of IE1 repression identified in this study have already been ascribed critical roles in the hCMV life cycle. To give but one example, IFNγ-inducible protein 16 (IFI16) acts as a foreign DNA sensor and restriction factor for hCMV [104, 105], suggesting that IE1 might promote viral replication by limiting its expression. In accordance with this speculation, replication of an IE1 mutant hCMV (TBIE1dl410-420) deficient for STAT3/STAT1 pathway diversion is attenuated compared to wild-type and revertant viruses. It should be noted, however, that the attenuated phenotype of the TBIE1dl410-420 virus does not necessarily result from repression of STAT3-responsive genes. In fact, this study mapped STAT3 binding between amino acids 410 and 445, while our previous work mapped STAT2 binding between amino acids 373 and 445 in IE1 [51]. Thus, STAT3 and STAT2 seem to bind to the same or closely adjoining sites in IE1, making it difficult to work out the extent by which either interaction contributes to the observed replication defect. That said, the replication defect exhibited by TBIE1dl410-420 in normal fibroblasts was entirely rescued in STAT2-deficient cells. Moreover, TBIE1dl410-420 proved to be more sensitive to exogenous IFNβ compared to wild-type and revertant viruses in normal fibroblasts. These observations indicate that the attenuation of TBIE1dl410-420 results from the lack of IE1-STAT2 interaction, which would normally promote viral replication by inhibiting type I IFN signaling. In contrast, the type II IFN-like response linked to IE1-STAT3 interaction appears to moderate rather than promote hCMV replication consistent with reports that IFNs govern viral latency, at least in murine cytomegalovirus (mCMV) [106, 107]. We are therefore tempted to speculate that, by imparting a low-level chronic IFNγ-like response, IE1 might contribute to viral quiescence in latently infected cells exposed to cytokines or growth factors that activate IL6ST. This idea adds to the emerging concept that IE1, a protein traditionally linked exclusively to the viral productive cycle, may also have an important role in hCMV latency [41, 101].

IL6 and IFNγ are among the most pivotal cytokines in shaping innate and adaptive host responses to infectious pathogens [7, 13]. In many instances, the two cytokines and their pathways have been associated with the outcomes of hCMV or mCMV infection and pathogenesis [30, 108–113]. In addition to IL6-type cytokines, many other important factors signal via the IL6ST-JAK1-STAT3 axis. These factors include GCSF and IL10, both of which have been linked to hCMV infection including viral latency and reactivation [114–116]. Signaling through STAT3 or STAT1 usually results in distinct and sometimes opposing outcomes. For instance, STAT1 tends to promote apoptosis in a variety of cell types whereas STAT3 typically has anti-apoptotic effects. Likewise, STAT1 usually acts anti-proliferative while STAT3 rather promotes cellular proliferation [14, 17]. By merging two major signaling pathways with diverse actions in many cell and tissue types, IE1 may impact hCMV infection and pathogenesis in surprising ways that future work will explore.

## Materials and Methods

### Plasmids

The pMD2.G and psPAX2 packaging vectors for lentivirus production were obtained from Addgene (plasmids #12259 and #12260, respectively). The lentiviral plasmid pLKOneo.CMV.EGFPnlsTetR, encoding the tetracycline repressor linked to a nuclear localization signal and the enhanced green fluorescent protein (EGFP) [117], was kindly provided by Roger Everett (University of Glasgow). Plasmids pCMV.TetO.cIE1 and pLKO.DCMV.TetO.cIE1 (herein referred to as pLKO.DCMV.TetO.IE1), expressing hCMV (Towne) IE1 under the control of a tetracycline- or dox-inducible promoter, have been described [21]. Variants of these plasmids encoding wild-type IE1 linked to an N-terminal hemagglutinin (HA) epitope tag (pLKO.DCMV.TetO.HA-IE1) or HA-tagged IE1 deletion mutants were constructed by standard PCR (IE1, IE1dl476-491, IE19, NLS-IE1dl1-404, 2×Stop-IE1) or overlap extension PCR (IE1dl373-386, IE1dl387-394, IE1dl395-409, IE1dl410-420, IE1dl421-445, IE1dl446-450 and IE1dl451-475) using oligonucleotide primers listed in Table 3. For each new construct, the entire IE1-specific nucleotide sequence was verified by DNA sequencing. The lentiviral plasmids used for inducible expression of human STAT3- or firefly luciferase-specific shRNAs (shSTAT3_1, shSTAT3_2 and shLUC) were generated by ligating annealed oligonucleotides (Table 3) to *Eco*RI- and *Age*I-digested Tet-pLKO-puro (Addgene plasmid #21915), and the resulting constructs were verified by DNA sequencing.

**Table 3:** Oligonucleotides used in this study.

#	Sequence (5‘→3‘)^1^	Use^2^
151	CTCTCCTAGTGTGGATGACC	PCR (pTBwt)
		PCR (pTBIE1dl410-420)
		PCR (pTBrvIE1dl410-420)
363	TATCAGCAGTACCAGGATGC	RT-qPCR (TUBB)
364	TGAGAAGCCTGAGGTGATG	RT-qPCR (TUBB)
386	TCCTAGTGTGGATGACCTACGGTACACTTTGGCCACCGCTGGTG	pCMV.TetO.HA-IE1dl373-386
387	CACCAGCGGTGGCCAAAGTGTACCGTAGGTCATCCACACTAGGA	pCMV.TetO.HA-IE1dl373-386
388	CACTTTGGCCACCGCTGGTGCCGCGACTATCCCTCTGTCCTCAG	pCMV.TetO.HA-IE1dl395-409
389	CTGAGGACAGAGGGATAGTCGCGGCACCAGCGGTGGCCAAAGTG	pCMV.TetO.HA-IE1dl395-409
390	TCTGTCCTCAGTAATTGTGGCTACTGTGTCTGTCAAGTCTGAGC	pCMV.TetO.HA-IE1dl421-445
391	GCTCAGACTTGACAGACACAGTAGCCACAATTACTGAGGACAGA	pCMV.TetO.HA-IE1dl421-445
392	GGAGGACACTGTGTCTGTCAAGGGAGGCAAGAGCACCCACCCTA	pCMV.TetO.HA-IE1dl451-475
393	TAGGGTGGGTGCTCTTGCCTCCCTTGACAGACACAGTGTCCTCC	pCMV.TetO.HA-IE1dl451-475
401	TGAGGAAGAGGCTATTGTAGCCAGCTCCTCTGATTCTCTGGTGT	pCMV.TetO.HA-IE1dl387-394
402	ACACCAGAGAATCAGAGGAGCTGGCTACAATAGCCTCTTCCTCA	pCMV.TetO.HA-IE1dl387-394
430	CTCACTACATGTGTGGAA	PCR (pgTBwt)
		PCR (pgTBIE1dl410-420)
		PCR (pgTBrvIE1dl410-420)
467	CTTGTGGCTGATGTGAATGC	RT-qPCR (IFI16)
468	AGGAGTTACGCTGGCACTTC	RT-qPCR (IFI16)
471	TCCCTAAGACCACCAATG	RT-qPCR (IE1)
472	GAGCACTGAGGCAAGTTC	RT-qPCR (IE1)
484	TTGCAGAATTCTTACTGGTCAGCCTTGCTTCTAG	pCMV.TetO.HA-NLS-IE1dl1-404
533	TCCACGTGTTGAGATCATTGC	RT-qPCR (CXCL10)
534	TCTTGATGGCCTTCGATTCTG	RT-qPCR (CXCL10)
535	CAAGGCTTCCCCATGTTCA	RT-qPCR (CXCL11)
536	CCCAGGGCGTATGCAAAGA	RT-qPCR (CXCL11)
537	GCTCCAAGCAGTCCTTTCAC	RT-qPCR (GBP4)
538	GTGGTGGCTCATGCCTAAAT	RT-qPCR (GBP4)
598	AGTGAGTAAGGCTGGGCAGA	RT-qPCR (STAT3)
599	AAGGCACCCACAGAAACAAC	RT-qPCR (STAT3)
672	CATCAATCATCACCCTGTGG	RT-qPCR (JAK1)
673	CCAGCTACCTCCAAGCAAAC	RT-qPCR (JAK1)
688	CTGGCGGCTATAAACCTAACC	RT-qPCR (OAS1)
689	GTTCTGTGAAGCAGGTGGAGA	RT-qPCR (OAS1)
690	ATGATGGAAAGCGAACAAGG	RT-qPCR (EIF2AK2)
691	CAGCAAGAATTAGCCCCAAA	RT-qPCR (EIF2AK2)
694	GATACTGAATTCTTACTGGTCAGCCTTGCTTCTAGT	pCMV.TetO.HA-IE1wt
		pCMV.TetO.HA-IE1dl373-386
		pCMV.TetO.HA-IE1dl387-394
		pCMV.TetO.HA-IE1dl395-409
		pCMV.TetO.HA-IE1dl410-420
		pCMV.TetO.HA-IE1dl421-445
		pCMV.TetO.HA-IE1dl446-450
		pCMV.TetO.HA-IE1dl451-475
		pCMV.TetO.HA-IE19
		pCMV.TetO.HA-2×Stop-IE1wt
695	GATACTGAATTCTTAAGAGGCGGTGGGTTCCTCAGCACC	pCMV.TetO.HA-IE1dl476-491
749	GGCCACTCTTCAGCATCTC	RT-qPCR (SOCS3)
750	ATCGTACTGGTCCAGGAACTC	RT-qPCR (SOCS3)
771	ACTTTCTTCCTGATCACTGTTCTCGGGTACAGGGGACTCTGGGGGTGA	pCMV.TetO.HA-IE1dl410-420
772	TCACCCCCAGAGTCCCCTGTACCCGAGAACAGTGATCAGGAAGAAAGT	pCMV.TetO.HA-IE1dl410-420
773	CTCTATCTCAGACACTGGCTCAGAGTCCTCCCGCTCCTCCTGAGCACC	pCMV.TetO.HA-IE1dl446-450
774	GGTGCTCAGGAGGAGCGGGAGGACTCTGAGCCAGTGTCTGAGATAGAG	pCMV.TetO.HA-IE1dl446-450
809	GATACTAAGCTTGCCACCATGTATCCTTACGACGTGCCTGACTACGCCGAGTCCTCTGCCAAGAGAAAGATG	pCMV.TetO.HA-IE1wt
		pCMV.TetO.HA-IE1dl373-386
		pCMV.TetO.HA-IE1dl387-394
		pCMV.TetO.HA-IE1dl395-409
		pCMV.TetO.HA-IE1dl410-420
		pCMV.TetO.HA-IE1dl421-445
		pCMV.TetO.HA-IE1dl446-450
		pCMV.TetO.HA-IE1dl451-475
		pCMV.TetO.HA-IE1dl476-491
		pCMV.TetO.HA-IE19
872	GCGTTTAATGTCGTCGCTCAA	qPCR (hCMV UL86)
873	CAGCCTACCCGTACCTTTCCA	qPCR (hCMV UL86)
880	AAACCTTTGAACAAGTGACCGAGGATTGCAACGAGAACCCCGAAAAAGATGTCCTGGCAGAACTCGGTAAGT	pTBrvIE1dl410-420
		pgTBrvIE1dl410-420
881	ATAAGAAGACACGGGAGACTTAGTACGGTTTCACAGGCGTGACACGTTTATTGAGTAGGATTACAGAGTATA	pTBrvIE1dl410-420
		pgTBrvIE1dl410-420
916	GATACTAAGCTTGCCACCATGTATCCTTACGACGTGCCTGACTACGCCTAGTGATCTGCCAAGAGAAAGATG	pCMV.TetO.HA-2×Stop-IE1wt
923	GATACTAAGCTTGCCACCATGTATCCTTACGACGTGCCTGACTACGCCCCCAAGAAGAAGAGAAAGGTGGAGTCCCCTGTACCCGCGACTATC	pCMV.TetO.HA-NLS-IE1dl1-404
927	GGCCGGTGCTACTGGAATCGATAC	PCR (pTBwt)
		PCR (pTBIE1dl410-420)
		PCR (pTBrvIE1dl410-420)
961	P-GCCACCATGGCCCAATGGAATCAGCTACAG	pLHCX-STAT3α-Myc
962	P-TCACAGATCCTCTTCTGAGATGAGTTTCTGCTCCATGGGGGAGGTAGCGCACTCCGA	pLHCX-STAT3α-Myc
1029	AGCCTTTCTCTGCTGCGAGT	ChIP (SOCS3 promoter)
1030	CCCGATTCCTGGAACTGC	ChIP (SOCS3 promoter)
1031	GGAATGTAGCAGCGATGGAA	ChIP (SOCS3 coding region)
1032	GCCCTGTCCAGCCCAATAC	ChIP (SOCS3 coding region)
1041	CAGGGTTTCAGGTTCCAATC	RT-qPCR (CXCL12)
1042	CACCAGGACCTTCTGTGGAT	RT-qPCR (CXCL12)
1043	ACGGCTTCCAGGTTAAGGTT	RT-qPCR (C4A)
1044	GTGCAGGACTTGGGTGATCT	RT-qPCR (C4A)
1047	AAGTAGGGTTCAGCCAAAGC	RT-qPCR (CHL1)
1048	ACGTGAACCTGTTCTACATACC	RT-qPCR (CHL1)
1049	TGTCATCATCGTGGGCAACA	RT-qPCR (RASL11A)
1050	CCAGCTCATTGGCTAGCTGA	RT-qPCR (RASL11A)
1051	GTAGCGCTGCCCCATTCCTGAAGACCAAG	pLHCX-STAT3α_Y705F-Myc
1052	CTTGGTCTTCAGGAATGGGGCAGCGCTAC	pLHCX-STAT3α_Y705F-Myc
1075	GCTGCTTGGGTTCTCCATAG	RT-qPCR (IL6ST)
1076	GCTCTGGCTTCGTATCTGTT	RT-qPCR (IL6ST)
1094	CTCTGATTCTCTGGTGTCACCCCCAGAGTCCCCTGTACCCGAGAACAGTGATCAGGAAGATAGGGATAACAGGGTAATCGATTT	pTBIE1dl410-420
		pgTBIE1dl410-420
1095	CCTCATCACTCTGCTCACTTTCTTCCTGATCACTGTTCTCGGGTACAGGGGACTCTGGGGGCCAGTGTTACAACCAATTAACC	pTBIE1dl410-420
		pgTBIE1dl410-420
1098	P-CCGGGTGCGTTGCTAGTACCAACCTCGAGGTTGGTACTAGCAACGCACTTTTT	Tet-pLKO-puro.shLUC
1099	P-AATTAAAAAGTGCGTTGCTAGTACCAACCTCGAGGTTGGTACTAGCAACGCAC	Tet-pLKO-puro.shLUC
1102	P-CCGGGCCTCAAGATTGACCTAGACTCGAGTCTAGGTCAATCTTGAGGCTTTTT	Tet-pLKO-puro.shSTAT3_1
1103	P-AATTAAAAAGCCTCAAGATTGACCTAGACTCGAGTCTAGGTCAATCTTGAGGC	Tet-pLKO-puro.shSTAT3_1
1134	P-CCGGAGTCAGGTTGCTGGTCAAACTCGAGTTTGACCAGCAACCTGACTTTTTT	Tet-pLKO-puro.shSTAT3_2
1135	P-AATTAAAAAAGTCAGGTTGCTGGTCAAACTCGAGTTTGACCAGCAACCTGACT	Tet-pLKO-puro.shSTAT3_2
1165	CTCTGGGGCAACTTCCTCTA	PCR (pgTBwt)
		PCR (pgTBIE1dl410-420)
		PCR (pgTBrvIE1dl410-420)
1291	TATTGAAAGCGGAGAAATGCC	RT-qPCR (MYOCD)
1292	GAAGATCTGGGTATCTTTGGGA	RT-qPCR (MYOCD)
1293	CACGATGTGAATTGGTTGGT	RT-qPCR (PDK4)
1294	TGCCTTTGAGTGTTCAAGGA	RT-qPCR (PDK4)
1295	AATCCTTGGAGCCCTAACAG	RT-qPCR (TMOD1)
1296	AGCAGTGCATTATCAGGGT	RT-qPCR (TMOD1)
1297	GCGTATGTGAAATTCGTCCCT	RT-qPCR (LRRN1)
1298	TCCTTGTTAAGCGGAGGTCAT	RT-qPCR (LRRN1)
1299	CTTCAAAGCTTTGGTCCAC	RT-qPCR (AKR1C3)
1300	CCAATTGAGCTTTCTTCAGTG	RT-qPCR (AKR1C3)
1301	AGAAAGGATTCCCTCCTCCA	RT-qPCR (RGS5)
1302	CAGCCAATCCAGAGCCTTAG	RT-qPCR (RGS5)
1303	ATCACATTCCAGCAGGCTTC	RT-qPCR (STC1)
1304	CCTGAAGCCATCACTGAGGT	RT-qPCR (STC1)
1305	CTGCTGCACATCGTCATTGAC	RT-qPCR (EDNRB)
1306	GCTCCAAATGGCCAGTCCT	RT-qPCR (EDNRB)
1307	GCATACAGGGACAACAAACAAC	RT-qPCR (PDE1A)
1308	TCTCAAGGACAGAGCGATCAT	RT-qPCR (PDE1A)
1309	ACAGGTCCAACCTACATGCC	RT-qPCR (TSPAN2)
1310	TGAGCTGGAGCTTAACACTGAT	RT-qPCR (TSPAN2)
1311	AGTATCGCTACTTCAAAGGGT	RT-qPCR (GPCPD1)
1312	CAACACCATTGTGGATTCCA	RT-qPCR (GPCPD1)
1315	GAAATGGTAATCCAGCTCCTG	RT-qPCR (PTGFR)
1316	AATGTTGGCCATTGTAACCAG	RT-qPCR (PTGFR)
1317	TGAAATGTAAATGGTCCTGTCG	RT-qPCR (HRSP12)
1318	GTCGTCCTTGATCAGAAGGG	RT-qPCR (HRSP12)
1325	TTCACAGGCTATCCAAAATTCA	RT-qPCR (RGS18)
1326	TTTGACAAACTGCTTTCCCA	RT-qPCR (RGS18)

^1^ P-, 5‘ phosphate.

^2^ RT-qPCR-based quantification of the indicated mRNA, qPCR-based hCMV or ChIP quantification or PCR-based construction of the indicated plasmid or BAC.

First strand cDNA of the full-length human STAT3α coding sequence was prepared from total RNA extracted from MRC-5 cells using an oligo(dT)_20_ primer. The STAT3α cDNA was linked to a C-terminal Myc epitope tag and PCR-amplified using oligonucleotide primers #961 and #962 (Table 3). The purified PCR product was ligated to *Hpa*I-digested, dephosphorylated retroviral vector pLHCX (Clontech, 631511) resulting in plasmid pLHCX-STAT3α-Myc. QuikChange site-directed mutagenesis of pLHCX-STAT3α-Myc using oligonucleotide primers #1051 and #1052 (Table 3) resulted in plasmid pLHCX-STAT3α_Y705F-Myc encoding a trans-dominant negative STAT3α-Myc variant resistant to phosphorylation at Y705 [66]. For both new constructs, the correct orientation and nucleotide sequence of the STAT3 insert were verified by DNA sequencing.

To generate expression plasmids for proteins linked to N-terminal mCherry, the mCherry cDNA was PCR-amplified from plasmid pIM-Asf1B-mCherry (kindly provided by Jean-Yves Thuret, Paris-Sud University) using oligonucleotide primers #1201 and #1202 (Table 3) and ligated to *Bgl*II- and *Hin*dIII-digested pCMV.TetO.HA-IE19. The HA-IE19 insert of the resulting construct was released with *Hin*dIII and *Eco*RI and replaced with the HA-IE1 coding sequences of plasmids pCMV.TetO.HA-IE1, pCMV.TetO.HA-2×Stop-IE1 and pCMV.TetO.HA-NLS-IE1dl1-404. The resulting constructs were verified by DNA sequencing.

Plasmid templates pLAY2 and pUC-MIE-Kan_I-SceI for generation of mutant and revertant hCMV TB40/E BACs by *en passant* mutagenesis, kindly provided by Karsten Tischer (Freie Universität Berlin), have been described [62, 94, 118].

### Antibodies

The primary antibodies used in this work were as follows: mouse anti-HA (Covance, MMS-101P), mouse anti-IE1 ([119], 1B12), mouse anti-IE1/IE2 (Millipore, MAB810R), mouse anti-Myc (Santa Cruz Biotechnology, sc-40), rabbit anti-glyceraldehyde 3-phosphate dehydrogenase (GAPDH) (Abcam, ab9485), rabbit anti-H2B (Abcam, ab1790), rabbit anti-STAT1 p84/p91 (Santa Cruz Biotechnology, sc-346), rabbit anti-STAT1α p91 (Santa Cruz Biotechnology, sc-345), rabbit anti-STAT2 (Santa Cruz Biotechnology, sc-22816), rabbit anti-STAT3 (Santa Cruz Biotechnology, sc-482x), rabbit anti-STAT3α (Cell Signaling Technologies, 8768), rabbit anti-pSTAT1 (Y701) (Cell Signaling Technologies, 9171), rabbit anti-pSTAT1 (S727) (Cell Signaling Technologies, 9177), rabbit anti-pSTAT3 (Cell Signaling Technologies, 9145), rabbit anti-SUMO1 (Santa Cruz Biotechnology, sc-9060) and mouse anti-α-tubulin (TUBA) (Thermo Fisher Scientific, A-11126).

The following secondary antibodies were used: peroxidase-conjugated goat anti-mouse immunoglobulin G (IgG) (Dianova, 115-035-166) or goat anti-rabbit IgG (Dianova, 111-035-144) for immunoblotting, and highly cross-adsorbed Alexa Fluor 488-conjugated goat anti-mouse IgG (Thermo Fisher Scientific, A-11001) or Alexa Fluor 594-conjugated goat anti-rabbit IgG (Thermo Fisher Scientific, A-11037) for immunofluorescence.

### Cells and retroviruses

MRC-5 human embryonic lung fibroblasts (American Type Culture Collection, CCL-171), STAT2-deficient primary skin fibroblasts from a 5-year-old child with a history of disseminated vaccine-strain measles [120] (kindly provided by Sophie Hambleton, Newcastle University) and Phoenix-Ampho retrovirus packaging cells (kindly provided by Garry Nolan, Stanford University) were maintained in Dulbecco's Modified Eagle’s Medium (DMEM) supplemented with 10% fetal calf serum (FCS), 100 units/ml penicillin and 100 μg/ml streptomycin. The human embryonic kidney cell line 293T (GenHunter, Q401) was cultured in the same medium containing 400 μg/ml G418 sulfate. All cultures were regularly screened for *Mycoplasma sp*. using an in-house qPCR assay. Where applicable, cells were treated with 1,000 U/ml recombinant human IFNα A/D (R&D Systems, 11200), 1,000 U/ml IFNβ1α (Miltenyi Biotec, 130-107-888), 1,000 U/ml recombinant human IFNγ (R&D Systems, 285-IF), 100 ng/ml recombinant human OSM (R&D Systems, 295-OM-010) or 100 ng/ml recombinant human IL6 (R&D Systems, 206-IL-010) plus 100 ng/ml recombinant human IL6Rα (R&D Systems, 227-SR-025) for the indicated durations. Fibroblasts are normally unresponsive to IL6, since they have little or no IL6R (also known as IL6Rα). However, soluble IL6R can bind IL6 with similar affinity as membrane-bound IL6R and the complex of IL6 and soluble IL6R can interact with and signal through IL6ST (also known as IL6Rβ or gp130).

Production of replication-deficient retroviral particles, retrovirus infections and selection of stable cell lines were performed as previously described [21] with minor modifications. Lentiviral particles were generated by transient transfection of 293T cells using calcium phosphate co-precipitation [121]. Recombinant viruses were collected 48 h after transfection and used for two consecutive transductions of 4 h each. To generate TetR cells, low-passage MRC-5 cells were transduced with pLKOneo.CMV.EGFPnlsTetR-derived lentiviruses and selected with G418 sulfate (0.3 mg/ml). To generate TetR-IE1 cells, TetR cells were transduced with pLKO.DCMV.TetO.HA-IE1-derived lentiviruses and selected with puromycin (1 μg/ml). TetR cells were maintained in medium containing G418 sulfate (0.3 mg/ml), while TetR-IE1 cells were cultured in the presence of both G418 sulfate (0.3 mg/ml) and puromycin (1 μg/ml). To induce IE1 expression, cells were treated with dox (Clontech, 631311) at a final concentration of 1 μg/ml. To generate cells with inducible expression of human STAT3- or firefly luciferase-specific shRNAs, MRC-5 cells were transduced with Tet-pLKO-puro.shSTAT3_1-, Tet-pLKO-puro.shSTAT3_2- or Tet-pLKO-puro.shLUC-derived lentiviruses and selected in medium containing puromycin (1 μg/ml). To induce shRNA expression, cells were treated with 1 μg/ml dox. At least half of the culture medium was replaced every 48 h with fresh dox added to maintain stable shRNA expression. To generate cells constitutively expressing STAT3α-Myc proteins, TetR cells were infected with pLHCX-derived retroviruses encoding STAT3α-Myc or STAT3α_Y705F-Myc and selected in medium containing hygromycin B (0.2 mg/ml).

### hCMV mutagenesis and infection

Wild-type virus of the low passage hCMV strain TB40/E (TBwt) was derived from TB40-BAC4 [122], kindly provided by Christian Sinzger (Ulm University). A modified version of this BAC with an SV40-EGFP-BGH PolyA cassette inserted between the US34 and TRS1 genes [123] was kindly provided by Felicia Goodrum (University of Arizona) and used to generate the EGFP expressing TB40/E wild-type virus gTBwt. Mutant BACs encoding IE1 with internal deletion of amino acids 410 to 420 (pTBIE1dl410-420 and pgTBIE1dl410-420) and corresponding revertant BACs (pTBrvIE1dl410-420 and pgTBrvIE1dl410-420) were generated from pTBwt or pgTBwt by markerless *en passant* mutagenesis, as previously described [62, 94] using plasmids pLAY2 and pUC-MIE-Kan_I-SceI and oligonucleotide primers listed in Table 3. The identity and integrity of each BAC were verified by DNA sequencing of the modified region and restriction fragment length analysis following digestion with *Eco*RI, respectively. Viruses were reconstituted and virus stocks produced upon electroporation of MRC-5 cells with BAC DNA following standard protocols. Titers were determined by plaque assay, and quantification of intracellular viral genome equivalents was performed as described [53] except that cells were cultured in the presence of phosphonoacetic acid (0.2 mg/ml) for 18 h before DNA isolation and qPCR analysis. MRC-5 lung and STAT2-deficient skin fibroblasts were grown to confluency, serum-starved in DMEM containing 0.5% FBS for 24 h and infected in medium with 0.5% FBS. After 6 h the inoculum was removed, cells were rinsed with pre-warmed DMEM and cultured in serum-reduced (0.5% FBS) DMEM.

### RNA interference

Silencer Select siRNAs (chemically modified, 21-mer, locked nucleic acid, double-stranded RNAs; Thermo Fisher Scientific) at a final concentration of 30 nM were introduced into cells using the Lipofectamine RNAiMAX Reagent (Thermo Fisher Scientific) and following the manufacturer’s protocols. Briefly, exponentially growing cells were seeded either in 12-well dishes at 2.5×10^5^ cells/well for RNA analyses or in 6-well dishes at 5×10^5^ cells/well for protein analyses. Transfections were performed in Opti-MEM I Reduced Serum Medium (Thermo Fisher Scientific) with 2 μl or 5 μl transfection reagent for 12 or 6 wells, respectively. The siRNA sequences are listed in Table 4.

**Table 4:** siRNAs used in this study.

#	Name	Sequence (5‘→3‘)^1^	Company (catalog no.)	Use
143	siSTAT3_1	UCUAGGUCAAUCUUGAGGCdCdT	Ambion (s743)	STAT3 knock-down
151	siSTAT3_2	AAUCUUAGCAGGAAGGUG CdCdT	Ambion (s745)	STAT3 knock-down
165	siJAK1_1	UUGUCAUCAACGGUGAUGGdTdG	Ambion (s7646)	JAK1 knock-down
166	siJAK1_2	UCCAUGAUGAGCUUAAUACdCdA	Ambion (s7647)	JAK1 knock-down
169	siIFNGR1_1	UACGAGUUUAAAGCGAUGCdTdG	Ambion (s7193)	IFNGR1 knock-down
170	siIFNGR1_2	UCAAUUGUAACAUUAGUUGdGdT	Ambion (s7194)	IFNGR1 knock-down
173	siIL6ST_1	UAAGAUACUAGACAGUUCCd TdC	Ambion (s7317)	IL6ST knock-down
174	siIL6ST_2	UAAUCAACAGUGCAUGAGGdTdG	Ambion (s7318)	IL6ST knock-down
149	siControl^2^	unknown 21-mer	Ambion (4390843)	non-targeting control

^1^ guide (antisense) strand; d, desoxy.

^2^ Silencer Select Negative Control No. 1 siRNA.

### RNA analyses

The transcriptome analysis of TetR and TetR-IE1 cells using Affymetrix Human Gene 1.0 ST Arrays has been described [21], and the complete set of data is accessible through Gene Expression Omnibus (National Center for Biotechnology Information), Series GSE24434 (http://www.ncbi.nlm.nih.gov/geo/query/acc.cgi?acc=GSE24434). To determine steady-state mRNA levels by RT-qPCR, total RNA was isolated from fibroblasts cultured in 12-well dishes using the RNeasy Mini Kit and RNase-Free DNase Set (Qiagen) according to the manufacturer’s protocols. First-strand cDNA was synthesized at 50°C using the AffinityScript Multiple Temperature cDNA Synthesis Kit and oligo(dT) primers (Agilent Technologies) starting with equal amounts of total RNA. First-strand cDNA was diluted 10-fold in sterile ultrapure water, and 5 μl were used for real-time PCR exactly as described in detail in previous publications [21, 30]. The sequences of oligonucleotide primers used for qPCR are listed in Table 3.

### Protein analyses

Preparation of whole cell protein extracts, sodium dodecyl sulfate (SDS)-polyacrylamide gel electrophoresis and immunoblotting were performed according to previously published protocols [53, 93]. Subcellular fractionation was done as described [21]. For indirect immunofluorescence microscopy, cells were seeded on high-precision cover glasses with thickness No. 1.5H (VWR, MARI 0117640) and processed as described [21]. Following immunostaining, samples were covered with ProLong Gold Antifade Mountant with DAPI (Molecular Probes, P36931), and deconvoluted images were acquired using a BZ-9000 Biorevo all-in-one fluorescence microscope (Keyence).

For SUMOylation analysis, about 7×10^6^ cells were lysed on ice in 500 μl buffer containing 50 mM Tris-HCl pH 7.5, 150 mM NaCl, 2 mM MgCl_2_, 0.5% (v/v) Igepal CA-630, 1% (v/v) Triton X-100, 1% (v/v) protease inhibitor cocktail III (Calbiochem), 1% (v/v) phosphatase inhibitor cocktail II (Calbiochem) and 5 μg/ml N-ethylmaleimide. After sonification in a Branson Sonifier 450 cup horn resonator (25 pulses at duty cycle 80% and output control 10), lysates were cleared by centrifugation (20,000×g, 25 min, 4°C) and incubation with 20 μl mouse IgG agarose (Sigma-Aldrich). Soluble material was reacted with 10 μl anti-HA agarose (Sigma-Aldrich) for 2 h at 4°C with gentle rotation. Immune complexes were washed three times in modified radioimmunoprecipitation (RIPA) buffer (50 mM Tris-HCl pH 8.0, 150 mM NaCl, 0.1% [w/v] SDS, 1% [v/v] Igepal CA-630, 0.5% [w/v] sodium deoxycholate) and once in nuclease reaction buffer (50 mM Tris-HCl pH 8.0, 2 mM MgCl_2_) before incubation with 25 U Benzonase nuclease (Novagen) in a volume of 100 μl for 30 min. After two additional washing steps in RIPA buffer, proteins were eluted by addition of 80 μl 1× sample buffer [124] and incubation for 10 min at 95°C.

For co-immunoprecipitation analyses involving dox-treated TetR-IE1 fibroblasts, resting cells of two 15-cm dishes were cross-linked by treatment with 1% (v/v) formaldehyde for 10 min at room temperature. Monolayers were washed twice in ice-cold serum-free DMEM, and cells were scraped in DMEM supplemented with 1% protease inhibitor cocktail III, pelleted by centrifugation (1,200×g, 10 min, 4°C) and lysed in 1.5 ml RIPA buffer supplemented with 1% protease inhibitor cocktail III. After sonification (30 pulses at duty cycle 80% and output control 10 in a Branson Sonifier 450 cup horn resonator), lysates were cleared by centrifugation (20,000×g, 30 min, 4°C). Supernatants were incubated with 150 μl Protein A Agarose/Salmon Sperm DNA slurry (Millipore) for 45 min at 4°C. After centrifugation (14,000×g, 10 min, 4°C), 700 μl supernatant were incubated with 10 μg IE1/IE2 8B1.2 antibody or normal mouse IgG for 16 h at 4°C with gentle rotation. Antigen-antibody complexes were isolated by addition of 60 μl Protein A Agarose/Salmon Sperm DNA slurry. Complexes were washed once in RIPA buffer, and nucleic acids were removed by incubation with 25 U Benzonase nuclease in 100 μl nuclease reaction buffer for 30 min at 4°C. After five additional washing steps in RIPA buffer, bound proteins were eluted by incubation in 45 μl 1× sample buffer for 10 min at 99°C.

For co-immunoprecipitations involving plasmid-transfected 293T cell cultures, cells of a 10-cm dish were harvested in PBS without prior formaldehyde cross-linking. Cells were pelleted by centrifugation (550×g, 8 min, 4°C) and resuspended in 0.5 ml CoIP lysis buffer (50 mM Tris-HCl pH 8.0, 150 mM NaCl, 10% (v/v) glycerol, 0.5% (v/v) Triton X-100) supplemented with 1% protease inhibitor cocktail III. Lysates were incubated on ice for 10 min and cleared by centrifugation (20,000×g, 30 min, 4°C). Supernatants were incubated with 25 μl Pierce Anti-HA Magnetic Beads (ThermoFisher Scientific, 88836) for 2 h at 4°C with gentle rotation. Complexes were washed five times in CoIP wash buffer (50 mM Tris-HCl pH 8.0, 150 mM NaCl, 0.1% (v/v) Igepal CA-630) and eluted by incubation in 50 μl 1× sample buffer for 5 min at 95°C.

ChIP coupled to qPCR was performed essentially as described [21, 30] with minor modifications. Briefly, cells were cross-linked for 15 min at 37°C. Sheared chromatin was centrifuged for 30 min to remove insoluble material, and the supernatant from 7×10^6^ cells was subjected to a pre-clearing step with 75 μl Protein A Agarose/Salmon Sperm DNA slurry for 30 min at 4°C with gentle rotation. Immunoprecipitations were carried out using 10 μg STAT3 C-20 antibody or normal rabbit IgG. Oligonucleotide primers used for subsequent qPCRs are listed in Table 3.

### Bioinformatic analysis

The Core Analysis function (default analysis settings) of the Ingenuity Pathway Analysis software application (Content version 24718999, Build version 366632M; Qiagen) was used to explore upstream regulators in the human transcriptome repressed by IE1 relative to an Affymetrix Human Gene 1.0 ST Array reference set. Only direct and indirect relationships where ‘confidence = Experimentally Observed’ were considered.

## Supporting Information

S1 Data Human genes significantly up- or down-regulated by IE1 in the TetR-IE1 cell model. The listed probe sets are linked to average changes of ≥1.5-fold (up-regulated probe sets) or ≤-1.5-fold (down-regulated probe sets) at a significance level of 0.001 (confidence interval, CI = 99.9%) in GeneChip analyses comparing dox-treated TetR-IE1 (TetR-IE1+) to dox-treated TetR (TetR+) cells or TetR-IE1+ to solvent-treated TetR-IE1 (TetR-IE1-) cells 24 h or 72 h post induction. Data were derived from three replicate GeneChips (#1-#3) and include robust multi-array average (RMA)-normalized log_2_ signals (single and mean values) with standard deviation (STDEV) and 99.9% CI as well as log_2_ signal ratios and mean fold changes. The lists were filtered for and do not include probe sets with average changes of ≥1.5-fold (up-regulated probe sets) or ≤-1.5-fold (down-regulated probe sets) in analyses comparing TetR+ to TetR- cells at the corresponding (24 h or 72 h) post induction time. Probe sets significantly up- or down-regulated in both comparisons (TetR-IE1+ vs. TetR+ and TetR-IE1+ vs. TetR-IE1- cells) at the same post infection time are bold-typed. The complete GeneChip data are accessible at Gene Expression Omnibus, Series GSE24434 (http://www.ncbi.nlm.nih.gov/geo/query/acc.cgi?acc=GSE24434).
(XLS)

S1 Fig The majority of human genes down-regulated by IE1 are STAT3 target genes. MRC-5 cells transduced to express inducible shRNAs targeting firefly luciferase (shLUC) or human STAT3 (shSTAT3_1 and shSTAT3_2) were treated with dox for 72 h. Relative mRNA levels were determined by RT-qPCR with primers specific for the indicated cellular genes. Results were normalized to TUBB, and means and standard deviations of biological triplicates are shown in comparison to shLUC cells (set to 1).
(EPS)

S2 Fig Residues 405–491 within the IE1 C-terminal domain are sufficient for STAT3 binding. 293T cells were transfected with plasmids encoding mCherry-HA, mCherry-HA-IE1 (wild-type), or mCherry-HA-NLS-IE1dl1-404 fusion proteins. At 48 h post transfection, whole cell extracts were prepared and subjected to immunoprecipitations with anti-HA magnetic beads. Samples of lysates and immunoprecipitates (IPs) were analyzed by immunoblotting for STAT3α and HA-tagged proteins.
(EPS)

S3 Fig Down-regulation of genes responsive to STAT3, IL6 or/and OSM precedes up-regulation of genes responsive to STAT1 or/and IFNγ by IE1. Maximum average expression changes in genes ≥1.5-fold down- or up-regulated by IE1 (based on S1 Data) and regulated by STAT3, IL6 or/and OSM or STAT1 or/and IFNγ, respectively (based on Ingenuity Pathway Analysis), are compared between 24 h and 72 h following the onset of IE1 expression.
(EPS)

S4 Fig Knock-down of IFNGR1 only modestly affects IE1-mediated induction of IFNγ-stimulated genes. TetR (w/o) or TetR-IE1 (IE1) cells were transfected with a control siRNA or two different siRNAs specific for IFNGR1. From 48 h post siRNA transfection, cells were treated with dox for 72 h. During the last 24 h of dox treatment, cells were treated with IFNγ or solvent. Relative mRNA levels were determined by RT-qPCR for IFNGR1, IE1 and the STAT1 target genes CXCL9, CXCL10 and CXCL11. Results were normalized to TUBB, and means and standard deviations of two biological and two technical replicates are shown in comparison to control siRNA-transfected cells (set to 1).
(EPS)

S5 Fig Characterization of recombinant TB40/E BACs. Restriction fragment length analysis of pTB- (A) or pgTB-derived (C) wt, IE1dl410-420 and rvIE1dl410-420 BACs (two independent clones each) after digestion of 1.2 μg DNA with *Eco*RI and separation in a 0.7% agarose-Tris-acetate-EDTA gel stained with ethidium bromide. 10 ng of pTB- (B) or pgTB-derived (D) wt, IE1dl410-420 and rvIE1dl410-420 BACs were PCR-amplified using IE1-specific oligonucleotide primers #151 and #927 (B) or #430 and #1165 (D), and PCR products were separated in a 1.0% agarose-Tris-acetate-EDTA gel stained with ethidium bromide.
(EPS)

S6 Fig Nuclear accumulation of STAT3 in hCMV-infected cells. (A) MRC-5 cells were mock-infected or infected with TBwt, TBIE1dl410-420 or TBrvIE1dl410-420 at high input multiplicity (5 PFU/cell). At 24 h post infection, subcellular localization of endogenous STAT3 was analyzed by indirect immunofluorescence microscopy. Samples were simultaneously reacted with a rabbit polyclonal antibody to STAT3 and a mouse monoclonal antibody to IE1, followed by incubation with a rabbit-specific Alexa Fluor 488 conjugate and a mouse-specific Alexa Fluor 594 conjugate. Cell nuclei were visualized by DAPI staining. Additionally, merge images of STAT3, IE1 and DAPI signals are presented. (B) Fluorescence intensities of STAT3 staining within the nucleus and cytoplasm were determined for 50 cells per each infection. The mean nuclear-to-cytoplasmic ratios (N/C) and standard deviations of the mean are shown.
(EPS)

S7 Fig IE1 rewires IL6 signaling to STAT1 activation also late in hCMV infection. (A) MRC-5 cells were mock-infected or infected with TBwt, TBIE1dl410-420 or TBrvIE1dl410-420 at high input multiplicity (5 PFU/cell). At 6 h post infection, cultures were treated with solvent or IL6 plus IL6R (IL6/Rα). At 72 h post infection, whole cell protein extracts were prepared and analyzed by immunoblotting for IE1/IE2, total STAT1, pSTAT1 (Y701) and GAPDH. (B) MRC-5 cells were mock-infected or infected with TBwt, TBIE1dl410-420 or TBrvIE1dl410-420 at high input multiplicity (5 PFU/cell). At 6 h post infection, cultures were treated with solvent or IL6 plus IL6R (IL6/Rα). At 72 h post infection, relative mRNA levels were determined by RT-qPCR for the STAT1 target gene CXCL9. Results were normalized to TUBB, and means and standard deviations of biological triplicates are shown in comparison to solvent-treated mock-infected cells (set to 1).
(EPS)

S8 Fig STAT3 knock-down inhibits replication of gTBwt and gTBIE1dl410-420. (A) MRC-5 cells transduced to express inducible shRNAs targeting firefly luciferase (shLUC) or human STAT3 (shSTAT3) were treated with dox for 72 h and then infected with gTBwt or gTBIE1dl410-420 at low input multiplicity (0.01 PFU/cell). Every 48 h, half of the culture media was replaced, and viral replication was assessed at day 7 post infection by fluorescence microscopy. (B) MRC-5 cells transduced to express inducible shRNAs targeting firefly luciferase (shLUC) or human STAT3 (shSTAT3) were treated with dox for 72 h and then infected with gTBwt or gTBIE1dl410-420 at low input multiplicity (0.01 PFU/cell). Every 48 h, half of the culture media was replaced, and viral replication was assessed at day 7 post infection by qPCR-based relative quantification of hCMV DNA from culture supernatants with oligonucleotide primers specific for the viral UL86 sequence. Data are presented as means and standard deviations from two biological and two technical replicates relative to gTBwt-infected shLuc cells (set to 1).
(EPS)
